# Comparative Bioinformatics and Experimental Analysis of the Intergenic Regulatory Regions of *Bacillus cereus hbl* and *nhe* Enterotoxin Operons and the Impact of CodY on Virulence Heterogeneity

**DOI:** 10.3389/fmicb.2016.00768

**Published:** 2016-05-24

**Authors:** Maria-Elisabeth Böhm, Viktoria M. Krey, Nadja Jeßberger, Elrike Frenzel, Siegfried Scherer

**Affiliations:** ^1^Lehrstuhl für Mikrobielle Ökologie, Zentralinstitut für Ernährungs- und Lebensmittelforschung, Wissenschaftszentrum Weihenstephan, Technische Universität München, FreisingGermany; ^2^Department of Veterinary Sciences, Faculty of Veterinary Medicine, Ludwig-Maximilians-Universität München, OberschleißheimGermany; ^3^Department of Molecular Genetics, Groningen Biomolecular Sciences and Biotechnology Institute, University of Groningen, GroningenNetherlands

**Keywords:** *Bacillus cereus*, enterotoxins, 5′ IGR, Nhe, Hbl, CodY, transcriptional regulation

## Abstract

*Bacillus cereus* is a food contaminant with greatly varying enteropathogenic potential. Almost all known strains harbor the genes for at least one of the three enterotoxins Nhe, Hbl, and CytK. While some strains show no cytotoxicity, others have caused outbreaks, in rare cases even with lethal outcome. The reason for these differences in cytotoxicity is unknown. To gain insight into the origin of enterotoxin expression heterogeneity in different strains, the architecture and role of 5′ intergenic regions (5′ IGRs) upstream of the *nhe* and *hbl* operons was investigated. *In silico* comparison of 142 strains of all seven phylogenetic groups of *B. cereus sensu lato* proved the presence of long 5′ IGRs upstream of the *nheABC* and *hblCDAB* operons, which harbor recognition sites for several transcriptional regulators, including the virulence regulator PlcR, redox regulators ResD and Fnr, the nutrient-sensitive regulator CodY as well as the master regulator for biofilm formation SinR. By determining transcription start sites, unusually long 5′ untranslated regions (5′ UTRs) upstream of the *nhe* and *hbl* start codons were identified, which are not present upstream of *cytK-1* and *cytK-2*. Promoter fusions lacking various parts of the *nhe* and *hbl* 5′ UTR in *B. cereus* INRA C3 showed that the entire 331 bp 5′ UTR of *nhe* is necessary for full promoter activity, while the presence of the complete 606 bp *hbl* 5′ UTR lowers promoter activity. Repression was caused by a 268 bp sequence directly upstream of the *hbl* transcription start. Luciferase activity of reporter strains containing *nhe* and *hbl* 5′ IGR *lux* fusions provided evidence that toxin gene transcription is upregulated by the depletion of free amino acids. Electrophoretic mobility shift assays showed that the branched-chain amino acid sensing regulator CodY binds to both *nhe* and *hbl* 5′ UTR downstream of the promoter, potentially acting as a nutrient-responsive roadblock repressor of toxin gene transcription. PlcR binding sites are highly conserved among all *B. cereus sensu lato* strains, indicating that this regulator does not significantly contribute to the heterogeneity in virulence potentials. The CodY recognition sites are far less conserved, perhaps conferring varying strengths of CodY binding, which might modulate toxin synthesis in a strain-specific manner.

## Introduction

*Bacillus cereus* is an opportunistic pathogen and food contaminant that produces several toxins causing gastrointestinal illness in humans. *B. cereus sensu stricto* is closely related to *B. thuringiensis* and *B. anthracis*, which are regarded to be one species on the basis of high core-genomic relatedness (Ash et al., 1991). Together with *B. mycoides, B. pseudomycoides, B. weihenstephanensis, B. toyonensis*, and *B. cytotoxicus* they form the *B. cereus sensu lato* group. Due to their diverse lifestyles, differences in plasmid content, and varying toxinogenic potentials, some strains of *B. cereus* are considered to be probiotics (Hong et al., 2005), while others are opportunistic pathogens, causing several human infections such as endophthalmitis (David et al., 1994), meningitis (Barrie et al., 1992) and periodontitis (Helgason et al., 2000) or foodborne illness (Stenfors Arnesen et al., 2008). Occasionally, *B. thuringiensis* strains were reported to be responsible for human infections resembling *B. cereus* infections (Damgaard et al., 1997; Kuroki et al., 2009). *B. cereus* spores are frequently detected in food originating from soil, dust and plant material. They are transferred through air as well as by cross-contamination from food and food-processing equipment (Frankland and Frankland, 1887; Daelman et al., 2013). Two different forms of food poisoning are recognized: Emesis is caused by ingestion of the small, cyclic and heat stable dodecadepsipeptide cereulide with a short incubation period of 0.5–6 h. The diarrheal type is caused by single or combined action of heat-labile enterotoxins acting on epithelial cells of the gastro-intestinal tract with an incubation period of 8–16 h (Shinagawa, 1990; Ehling-Schulz et al., 2004). The three most important pore-forming *B. cereus* cytotoxins that have been linked to diarrheal disease are hemolysin BL (Hbl), non-hemolytic enterotoxin (Nhe) and cytotoxin K (CytK). Hbl consists of a binding component B and the two lytic components L_1_ and L_2_. These proteins are encoded in one operon *hblCDAB* by the genes *hblA*, *hblD*, and *hblC*, respectively (Guinebretiere et al., 2002). Nhe is a tripartite toxin encoded by the operon *nheABC* and acts cytolytic on erythrocytes and epithelial cells due to its ability to form pores in the plasma membrane (Fagerlund et al., 2008). All three components NheA, NheB, and NheC are required for full toxic activity, although NheC is only expressed in small amounts due to translational repression (Lindbäck et al., 2004). The third diarrhea causing agent is the single-component toxin CytK (34 kDa), which is a hemolytic and dermonecrotic β-barrel pore-forming enterotoxin (Lund et al., 2000).

It was shown that toxicity varies greatly between strains and that the sole presence of enterotoxin genes is not sufficient for the classification of a *B. cereus* strain as being pathogenic or apathogenic (Dietrich et al., 2005; Jeßberger et al., 2015). Indeed, enterotoxin expression is highly complex and strain-specifically regulated (Jeßberger et al., 2015). The promoter regions of both *nhe* and *hbl* were described to harbor binding sites for a variety of transcriptional regulators such as Fnr, ResD, SinR, CcpA and PlcR, which supposedly control enterotoxin expression in *B. cereus* in a concerted action (Esbelin et al., 2008, 2009; Gohar et al., 2008; van der Voort et al., 2008; Fagerlund et al., 2014). These regulators control the expression of either broad or narrow-spectrum regulons responding to oxygen tension (ResD, Fnr), carbohydrate availability (CcpA) and to the *B. cereus* group specific quorum sensing peptide PapR (PlcR). However, enterotoxin expression is additionally regulated by nitrogen source availability and the general energetic cell status, sensed by the pleiotropic regulator CodY (Frenzel et al., 2012). CodY is a global regulator of adaptation to unfavorable environments, sensing nutrient availability through interaction with GTP and the BCAAs isoleucine, leucine and valine (Ratnayake-Lecamwasam et al., 2001; Shivers and Sonenshein, 2004; Sonenshein, 2005). In the majority of low GC gram-positive bacteria, the CodY regulon controls profound cellular functions, such as motility, chemotaxis, catabolism, production of proteases and virulence (Shivers and Sonenshein, 2004; Sonenshein, 2005; Handke et al., 2008; Majerczyk et al., 2010). In *B. cereus*, CodY indirectly controls the expression of virulence genes via the activation of the PlcR regulon (Frenzel et al., 2012; Lindbäck et al., 2012; Slamti et al., 2015), which includes the enterotoxin operons *nhe*, *hbl*, and *cytK* (Agaisse et al., 1999; Lund et al., 2000).

To decipher the basis of differing enterotoxin expression potentials, we compared 5′ IGRs of the most prominent enterotoxins (Nhe, Hbl, and CytK) by means of bioinformatics and *in vitro* protein-DNA binding experiments. The contribution of the strikingly long stretches of 5′ IGRs preceding the *nhe* and *hbl* operons to enterotoxin expression was studied in detail with consecutively trimmed 5′ IGR*-luxABCDE* fusions. We provide evidence that the *hbl* 5′ UTR naturally attenuates promoter activity, while the entire *nhe* 5′ UTR is necessary for maximal promoter activity. Our results show that CodY may modulate *hbl* and *nhe* expression by acting as a downstream roadblock repressor of toxin gene transcription in a strain-dependent manner.

## Materials and Methods

### Microbial Strains

In order to compare intergenic regulatory regions upstream of the enterotoxin operons *hbl*, *nhe*, and *cytK* strains representative for the seven phylogenetic groups were selected from a set of 142 *B. cereus sensu lato* strains (Böhm et al., 2015; **Table 1**). Out of these, 29 *nhe*, 23 *hbl*, 3 *cytK-1*, and 15 *cytK-2* 5′ IGRs were examined. Enterotoxin promoter activity was studied experimentally in *B. cereus* INRA C3 (IV) and gel mobility shift assays were performed with both *B. cereus* INRA C3 and *B. cytotoxicus* CVUAS 2833 (VII).

**Table 1:** List of representative *B. cereus sensu lato* strains used for comparison of intergenic regions.

		Analysis of intergenic regulatory region			
Cluster	Strain name	*nhe*	*hbl*	*cytK-1*	*cytK-2*
II	*B. cereus* BAG6X1-1		X		
II	*B. cereus* MHI 226	X			
II	*B. cereus* 14294-3 (M6)	X	X		X
II	*B. cereus* BAG5X2-1		X		
II	*B. cereus* BAG2O-3	X			
II	*B. cereus* RIVM BC 126	X	X		
III	*B. cereus* NVH 0075-95	X			
III	*B. cereus* HWW 274-2	X			X
III	*B. cereus* F4810/72	X			
III	*B. anthracis* Ames Ancestor	X			
III	*B. thuringiensis* s. finitimus YBT-020		X		
III	*B. thuringiensis* s. pulsiensis BGSC 4CC1				X
III	*B. cereus* F837/76	X	X		
III	*B. cereus* MHI 86	X			X
III	*B. cereus* SDA KA 96	X	X		X
III	*B. cereus* F528/94	X	X		
III	*B. cereus* F3162/04	X			
IV	*B. cereus* ATCC 14579	X	X		X
IV	*B. thuringiensis* Bt407	X			
IV	*B. thuringiensis* HD-771	X	X		
IV	*B. thuringiensis* IBL 200				X
IV	*B. thuringiensis* s. berliner ATCC 10792	X	X		X
IV	*B. thuringiensis* s. huazhongensis BGSC 4BD1		X		
IV	*B. cereus* VD014				X
IV	*B. cereus* VD156	X	X		
IV	*B. cereus* BAG1X2-2				X
IV	*B. cereus* #17	X	X		X
IV	*B. cereus* RIVM BC 964	X			
IV	*B. cereus* INRA C3	X	X		X
IV	*B. cereus* 6/27/S	X	X		X
IV	*B. cereus* F3175/03	X	X		
IV	*B. bombysepticus* str. Wang				X
V	*B. cereus* Rock3-28	X	X		
V	*B. cereus* Rock4-18				X
V	*B. thuringiensis* MC28		X		
V	*B. cereus* VD115	X			
V	*B. toyonensis* BCT-7112	X	X		
VI	*B. weihenstephanensis* WSBC 10204	X	X		
VI	*B. mycoides* DSM 2048	X	X		
VI	*B. cereus* BAG5X1-1	X	X		
VII	*B. cytotoxicus* NVH 391-98			X	
VII	*B. cytotoxicus* CVUAS 2833			X	
VII	*B. cytotoxicus* NVH 883/00			X	

*All strains possess the *nhe* operon. Strains marked by an X are included in the respective comparative analysis. The *nhe* 5′ IGR of clusters I and VII strains differs from those of other strains, which interfered with the sequence alignment. They were excluded from the *nhe* 5′ IGR alignment. The cluster I strains investigated do not possess *hbl* or *cytK* and are therefore not part of this analysis.*

### Bioinformatic Analyses

Multiple sequence alignments were computed using ClustalΩ^1^ and sequence conservation was graphically represented as sequence logo^2^. The web-based program ORF Finder^3^ was used to detect potential open reading frames (ORFs) embedded in the 5′ UTRs of enterotoxin genes. The 5′ UTR RNA sequences were further analyzed for similarity to known RNA families with Rfam v.12.0 (Nawrocki et al., 2015) and potential RNA secondary structures were predicted at default settings (linear RNA, 37°C, 1 M NaCl) with Mfold v.4.6 (Zuker, 2003).

### Media and Growth Conditions

All cloning steps were performed in *Escherichia coli* TOP10 grown in lysogeny broth (LB: 5 g/l yeast extract, 10 g/l tryptone, 10 g/l sodium chloride) at 150 rpm or on LB agar plates at 37°C. *B. cereus* INRA C3 was grown in modified MOD (Frenzel et al., 2012) or CGY medium (Beecher and Wong, 1994) at 30°C unless stated otherwise. When appropriate, cultures (16 h, 150 rpm) were supplemented with 120 μg/ml ampicillin or 5 μg/ml chloramphenicol. Modified MOD medium was used for the determination of promoter activity. Stock solutions of 2 M glucose and trace elements were prepared with ddH_2_O, filter sterilized (0.22 μm pore size), and added to the MOD medium to a final concentration of 20 mM. To obtain MOD + 1% CAAs or MOD + 1% tryptone, 10 g/l CAA, or 10 g/l tryptone were dissolved and autoclaved together with the MOD medium components (110°C, 10 min). CGY medium was prepared in a volume of 900 ml ddH_2_O. After autoclaving 100 ml of filter sterilized glucose were added to a final concentration of 1%. The media compositions are listed in detail in Supplementary Table S1.

### Determination of Transcription Start Sites with 5′ RACE

*Bacilli* were cultured in CGY medium supplemented with 1% (w/v) glucose as described previously (Jeßberger et al., 2015) to an OD_600_ of 4. Six milliliter of the cultures were harvested (10.000 g, 4°C, 10 min), cell pellets were snap-frozen in liquid nitrogen and stored at -80°C. Total RNA was extracted by resuspending the pellet in 1 ml TRIreagent (Sigma-Aldrich) followed by cell disruption using a Fastprep 24 instrument (M. P. Biomedicals, 0.1 mm zirconia beads). DNase I digestion and RNA isolation were performed as previously described (Dommel et al., 2010). DNA-free RNA was used as template for 5′ RACE (5′ RACE system for rapid amplification of cDNA ends, version 2.0, Invitrogen). Gene specific primers to detect TSSs of *hbl* and *nhe* are listed in Supplementary Table S2.

### Construction of Bioluminescent *B. cereus* Reporter Strains

To study gene regulation and promoter activities, bioluminescent *B. cereus* reporter strains were constructed. The promoter region of interest was inserted into the *E. coli/Bacillus* shuttle vector pXen1 (Francis et al., 2001), which contains the luciferase gene cassette *luxABCDE* by using primers comprising restriction sites for EcoRI and BamHI (Supplementary Table S2). Fragments were amplified from genomic DNA using a proof reading *Pfu* polymerase (Promega). The resulting plasmid was propagated in non-methylating *E. coli* INV110 and introduced in *B. cereus* by electroporation as described previously (Ehling-Schulz et al., 2005). Promoter fusions were verified by amplification of the insertion region with primers pXen for and pXen rev (Supplementary Table S2) and by sequencing (GATC Biotech).

Promoter fusions containing internal deletions were constructed by amplification of two regions adjacent to the target region for deletion by introducing XmaI restriction sites up- and downstream of the target region using the primer pairs A-B and C-D (Supplementary Table S2). After digestion and ligation of the two fragments AB and CD, a nested PCR with primers containing restriction sites for EcoRI and BamHI (Nhe for/Nhe rev or Hbl for/Hbl rev, respectively) allowed directional insertion of the promoter regions containing internal deletions into pXen1 as described above.

### Luciferase Assay

Enterotoxin promoter activity of *B. cereus* strains grown in different media at 37°C was monitored by detecting the luminescence signals at 490 nm using the Victor3^TM^ multilabel plate reader (Perkin Elmer). Precultures grown for 16 h at 150 rpm were diluted 1:1000 in their respective medium supplemented with 5 μg/ml chloramphenicol, distributed into a 96-well microtiter plate (μClear white, Greiner Bio-One) and incubated at 800 rpm and 37°C. Each condition was tested in quadruplicates and for each strain three independent biological replicates were analyzed. Cell density (OD_600_) and luminescence (490 nm, 0.1 s) were measured in hourly intervals for 19 h to detect the maximal promoter activity. Significance of differences between measured activities was calculated in the R free statistical software (version 3.1.1)^4^ using Welch’s *t*-test.

### CodY Overexpression and Purification

Heterologous expression of CodY from *B. cereus* INRA C3 and *B. cytotoxicus* CVUAS 2833 was performed in *E. coli* BL21(DE3) as a soluble N-terminal His_6_-tag fusion protein using the plasmid pET28b(+) as described previously (Frenzel et al., 2012). The primers CodY-C3-for, CodY-C3-rev, CodY-CVUAS-for and CodY-CVUAS-rev (Supplementary Table S2) containing restriction sites for NdeI and XhoI, respectively, were used to construct the overexpression plasmid.

Protein expression was induced in LB medium at an OD_600_ of 0.6 with 1 mM IPTG and cells were harvested after 5 h. Cell disruption and protein purification were performed as described (Frenzel et al., 2012) with the exception of using the Äkta purifier (Amersham Biosciences) with a Frac-950 fractionator. A step-wise elution was performed by increasing imidazole concentration from 10 mM to 83.5 mM, 304 mM, and 402 mM to a final concentration of 500 mM. CodY-containing fractions were pooled and then dialyzed and concentrated in buffer BS using ultrafiltration columns with a 10 kDa cut-off (Amicon Ultra-15, Merck Millipore). Protein purity was analyzed on a 15% SDS-polyacrylamide gel with Coomassie staining.

### Electrophoretic Mobility Shift Assays

DNA fragments containing the promoter regions P*cytK-2* (*B. cereus* INRA C3), P*cytK-1* (*B. cytotoxicus* CVUAS 2833, which is identical to P*cytK-1* of the type strain *B. cytotoxicus* NVH 391-98), parts of P*hbl* (*B. cereus* INRA C3) and P*nhe* (*B. cereus* INRA C3 and *B. cytotoxicus* CVUAS 2833) were amplified by PCR. Primer pairs and fragments ranging from 241 to 568 bp are listed in Supplementary Table S3. A fragment of the 16S rRNA gene *rrn* was used as negative control, since it lacks any similarity to the CodY consensus sequence. Electrophoretic mobility shift assays were performed as described previously (Frenzel et al., 2012) at 4°C with varying amounts of protein and 100 ng of target DNA. The molarity of DNA fragments is given in Supplementary Table S3. The equilibrium dissociation constant K_D_ was estimated on the basis of three independent replicates of each electrophoretic mobility analysis as described earlier (Belitsky and Sonenshein, 2011b).

## Results and Discussion

### Length of 5′ Intergenic Regions (5′ IGRs)

**FIGURE 1:**
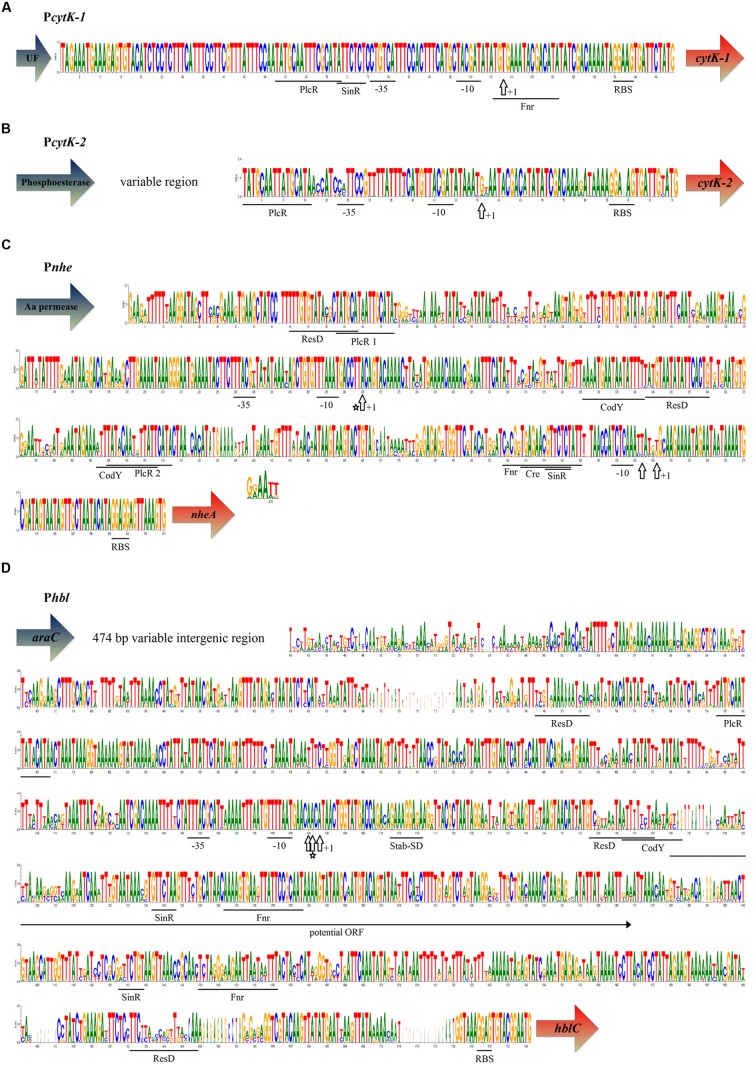
**Structure and sequence of intergenic regions containing enterotoxin promoters in *Bacillus cereus sensu lato*.** Sequence motifs were determined by sequence comparison. Promoter regions (-35, -10) and TSSs (+1, vertical arrow; Agaisse et al., 1999; Lindbäck et al., 1999, 2004; Brillard and Lereclus, 2004), CodY binding sites (den Hengst et al., 2005; Wray and Fisher, 2011; Frenzel et al., 2012; Belitsky and Sonenshein, 2013), catabolite responsive element (Cre; van der Voort et al., 2008), PlcR binding sites (Agaisse et al., 1999; Brillard and Lereclus, 2004; Lindbäck et al., 2004; Gohar et al., 2008), ribosomal binding site (RBS) of *hbl* (Ryan et al., 1997), ResD and Fnr binding sites (Geng et al., 2007; Esbelin et al., 2008, 2009), SinR binding sites (Kearns et al., 2005; Chu et al., 2006), and stabilizing Shine-Dalgarno sequence (Stab-SD; Agaisse and Lereclus, 1996). Conservation of the sequences is depicted as logo and based on a multiple sequence alignment of strains representative for the seven phylogenetic groups (see **Table 1** and Böhm et al., 2015). TSSs in *B. cereus* INRA C3 are marked by an asterisk. Colored arrows indicate gene function and transcriptional direction of genes. *AraC*: AraC family transcriptional regulator TrrA. Black arrow: transcriptional direction of a potential open reading frame (ORF). **(A)** *CytK-1* 5′ IGR of 3 *B. cytotoxicus* strains. Cluster VII strains contain a non-annotated ORF upstream of *cytK-1* (unknown function: UF). **(B)** *CytK-2* 5′ IGR of 15 *B. cereus sensu lato* strains. All strains are affiliated to phylogenetic clusters II–V. With the exception of the promoter, the intergenic region upstream of *cytK-2* is not conserved. 80% of all *cytK-2* strains possess a phosphoesterase gene directly upstream of *cytK-2*. In the remaining strains an insertion of 500–2000 bp length separates the two genes. **(C)** *Nhe* 5′ IGR of 29 *B. cereus sensu lato* strains. All clusters except I and VII contain a hypothetical amino acid permease gene upstream of *nheA*. The cluster VII (*B. cytotoxicus*) intergenic region contains the same promoter elements as the other strains with an overall sequence identity of 70–90%, but is ~50 bp shorter. The intergenic region of cluster I strains (*B. pseudomycoides*) consists of the same promoter elements, but is ~350 bp longer. Clusters I and VII strains were excluded from the analysis due to distortion of the multiple sequence alignment. **(D)** *Hbl* 5′ IGR of 23 *B. cereus sensu lato* strains. In 95% of all investigated *hbl* strains *araC* appears 1600–1200 bp upstream of *hblC*. The intergenic region upstream of *hbl* varies in size. Presented is the entire region of which up to 500 bp are lacking in several strains. Insertions occur in clusters II, V, and VI (nucleotides 1182–1192, 1651–1661) and in strains *B. weihenstephanensis* WSBC 10204 (nucleotides 696–720, 1706–1720) and *B. cereus* BAG5X1-1 (nucleotides 696–720, 1604–1610). A putative ORF starting with an alternative start codon (in most strains TCA or TAT) is noted. The *hbl* operon is part of a degraded, in most cases no longer functional transposon (Böhm et al., 2015). As a remnant, a transposase (pseudogene) occurs in two cluster VI strains (*B. cereus* BAG5X1-1) instead of *araC*.

To investigate the origin of differences in the regulation of enterotoxin expression, the 5′ IGRs of *cytK-1, cytK-2*, *nhe* and *hbl* of 142 *B. cereus sensu lato* strains (Böhm et al., 2015) were compared in a multiple sequence alignment approach (data not shown). Out of these, 29 *nhe*, 23 *hbl*, 3 *cytK-1*, and 15 *cytK-2* toxin genes of *B. cereus* strains representing the diversity of the seven phylogenetic groups of *B. cereus sensu lato* (Guinebretiere et al., 2008; Böhm et al., 2015) were selected and compared (**Figure 1**).

The promoter containing intergenic regions of *cytK-1* and *cytK-2* (**Figures 1A,B**) were found to be relatively short (~100 bp) in comparison to the *nhe* and *hbl* 5′ IGRs. *CytK-1* is exclusively found in rare *B. cytotoxicus* isolates, the most distant cluster of species within the *B. cereus* group (Guinebretiere et al., 2013). However, despite the limited strain number, the high similarity of 5′ *cytK-1* IGRs analyzed in this study is in agreement with the potentially highly clonal structure of this species (Guinebretiere et al., 2013). The *cytK-2* 5′ IGR, in contrast, consists of a differently sized variable region and a highly conserved promoter.

Some strains contain short insertions within their *nhe* 5′IGR, such as *B. pseudomycoides* DSM 12442 and other strains of the phylogenetic cluster I, which possess insertions downstream of each PlcR binding site (**Figure 1C**). The *nhe* 5′ IGR is approximately 350 bp longer in *B. pseudomycoides* strains compared to that of all other *B. cereus* group strains investigated. In contrast, *B. cytotoxicus* (cluster VII) contains an *nhe* 5′ IGR which is ~50 bp shorter than the other 5′IGRs, thus lacking the second PlcR binding site (data not shown). *B. weihenstephanensis* WSBC 10204 (cluster VI) contains a 14 bp insertion upstream of the *hbl* ribosomal binding site (**Figure 1D**). Strains of clusters III and IV, which harbor many pathogenic *B. cereus* isolates, lack 11 bp close to each ResD binding site within the *hbl* 5′ IGR. These missing regions might be used as an additional target to discern cluster III and IV from other *B. cereus* strains by molecular techniques. However, there is no correlation to high toxin production, since both high and low enterotoxigenic strains were found to harbor this deletions (Jeßberger et al., 2015). Taken together, all 5′ IGRs comprise well-conserved regions, but lengths of *hbl* and *cytK-2* 5′ IGRs are highly variable. With the exception of *B. pseudomycoides*, the insertions or deletions within 5′ IGRs are not specific at the species level.

### Transcription Start Sites and 5′ Untranslated Regions (5′ UTRs)

The TSSs of *nhe* in *B. cereus* strains NVH 0075-95 and NVH 1230-88 are localized 66 and 62 bp, respectively, upstream of the *nheA* startcodon (Lindbäck et al., 2004), while in *B. thuringiensis* Bt407 the TSS was reported to be located 331 bp upstream of *nheA* (Agaisse et al., 1999). This might indicate strain-specific differences in the promoter architecture. However, a 5′ RACE analysis revealed that the TSS of the *nhe* operon in *B. cereus* INRA C3 is identical with the TSS in *B. thuringiensis* Bt407 (**Figure 1C**). The 5′ UTR is approximately 350 bp long and shows a high degree of sequence similarity to all other analyzed *nhe* 5′ UTRs.

The TSS of the *hbl* operon in *B. cereus* ATCC 14579 was reported to localize 606 bp upstream of *hblC* (Lindbäck et al., 1999), which is identical to the transcription start of *hblC* in *B. cereus* INRA C3 (our data, **Figure 1D**). The TSS of the *hbl* cluster in *B. thuringiensis* Bt 407 is located 605 bp upstream of *hblC* (Agaisse et al., 1999). Thus, the 5′ IGR of *hbl* comprises an exceptionally long, generally conserved 5′ UTR of approximately 660 bp.

This study shows for the first time that the *nhe* and the *hbl* toxin operons are preceded by extended and widely conserved 5′ UTRs in their upstream intergenic sequences.

### Binding Sites of Transcriptional Regulators

Many regulator binding sites within the promoter regions P*nhe*, P*hbl*, and P*cytK* have been confirmed by experimental studies in individual strains during recent years. In this study, putative recognition sites of all known transcriptional regulators demonstrated so far to be involved in *B. cereus* enterotoxin expression were predicted by 5′ IGR alignments (**Figure 1**). In the *nhe* and *hbl* promoter regions putative binding sites for an overlapping set of transcription factors were identified. Two PlcR binding sites are localized within the *nhe* 5′ IGR. PlcR 1 is 16 bp long and highly conserved, while the less conserved PlcR binding site 2 contains a 2 bp central insertion compared to the consensus sequence TATGNAN_4_TNCATA (Gohar et al., 2008). Our sequence comparison-based analysis additionally predicted the stabilizing Shine-Dalgarno sequence in P*hbl* and putative binding sites for the master regulator of biofilm formation SinR in P*cytK-1*, P*nhe*, and P*hbl*. In contrast to the tripartite enterotoxin operons *nhe* and *hbl*, *cytK-1* and *cytK-2* are not preceded by 5′ UTRs. Apart from the detection of the PlcR binding site immediately upstream of the -35 element no further transcription factor binding site was described so far (Lund et al., 2000; Brillard and Lereclus, 2004). Our bioinformatical analysis of the 5′ IGR of *cytK-1* and *cytK-2* predicted putative SinR and Fnr binding motifs in P*cytK-1*. It must, however, be mentioned that an *in silico* analysis of regulator binding sites is always putative and, finally, needs experimental confirmation.

While *cytK* is expressed independently of CcpA-mediated catabolite control (van der Voort et al., 2008), the expression of *nhe* and *hbl* is regulated by carbon catabolite repression (the *cre* box of *hbl* is located downstream of the start codon). Our analysis additionally revealed that *nhe* and *hbl* 5′ IGRs contain motifs which may be relevant for *in vivo* binding of CodY (for further analysis and discussion see below).

### Other Potential Functions of 5′ IGRs

There is increasing evidence that intergenic regions in prokaryotes code for unknown small proteins (Neuhaus et al., 2016). Considering the size of several 100 bp, it is possible that the intergenic sequences upstream of *nhe* and *hbl* might encode small proteins or peptides. We could not detect any obvious ORFs upstream of *nhe*, but the 5′ UTR of *hbl* contains a putative ORF of varying size (180–192 nucleotides, **Figure 1D**). This ORF appears in all clusters, but not in all investigated strains, and is thus not restricted to particular phylogenetic groups. A BLASTP analysis did not reveal any homology to proteins of known function, thus the expression and function of this ORF remains to be studied.

5′ UTRs occasionally contain temperature sensitive RNA thermometers (Narberhaus et al., 2006) or metabolite responsive riboswitches Winkler and Breaker (2005). In *Listeria monocytogenes* long 5′ UTRs were found to regulate virulence gene expression, e.g., the *prfA* 5′ UTR is a thermosensor allowing transcription of the transcriptional activator at 37°C and blocking it at lower temperatures (Johansson et al., 2002; Loh et al., 2006). The activating and temperature-independent function of listerial *actA* and *hly* 5′ UTRs was shown (Wong et al., 2004; Shen and Higgins, 2005), but the mechanism of expression enhancement is not clear yet.

Recently, several repeat regions that might encode novel riboswitches have been identified in *B. cereus* (Kristoffersen et al., 2011), but none of them is located in the 5′ UTR of the *nhe* or *hbl* operons. Our Rfam analysis of the 5′ UTR sequences revealed no similarities with known RNA families and no conserved RNA secondary structures (data not shown). Nonetheless, the long 5′ IGRs could encode yet unknown functions or they may interact with different regulators to allow for differential expression of enterotoxin genes.

### Entire *nhe* 5′ IGR is Necessary for Maximal Promoter Activity

Our further experimental analysis focused on *B. cereus* INRA C3 due to the presence of all three main enterotoxins *nheABC, hblCDAB*, and *cytK-2* in this highly toxic strain (Jeßberger et al., 2015). For a detailed analysis of the regulator recognition sites in *B. cereus* INRA C3 *nhe* and *hbl* 5′ IGRs see Supplementary Figure S1.

**FIGURE 2:**
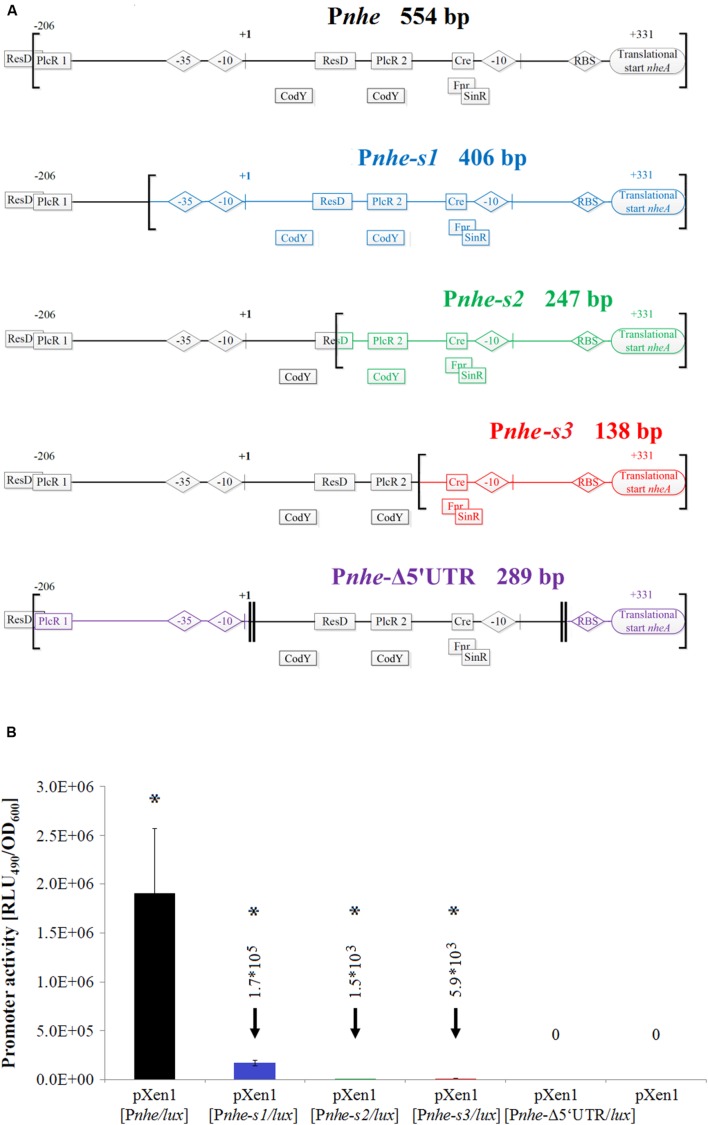
**Promoter activity of complete and partial *nhe* 5′ IGR in *B. cereus* INRA C3. (A)** P*nhe* full construct and shortened variants. Regions analyzed in promoter fusions are named and indicated by brackets, double lines enclose deletions and +1 is the TSS determined by 5′ RACE. Promoter elements and (putative) binding sites of transcriptional regulators (compare **Figure 1**) are displayed. **(B)** P*nhe* promoter activities were determined in modified MOD minimal medium in triplicates and compared at the time of peak activity of the construct containing the entire promoter region. Detailed growth and transcription kinetics of *B. cereus* INRA C3 P*nhe* promoter fusions are shown in Supplementary Figure S2A. Luminescence signals were generated by the transcription of *lux* genes located downstream of the complete or partial 5′ IGR tested for promoter activity. Statistically significant differences in transcriptional activity are marked by an asterisk (*p* < 0.05).

The functionality of the unusually long *nhe* 5′ UTR was investigated using several partial deletions of 5′ UTR sequences and luciferase as a reporter (**Figure 2A**). The promoter activities were assessed in the *B. cereus* INRA C3 background. The *nhe* operon was reported to contain two promoters and two PlcR binding sites (**Figures 1C** and **2A**; Agaisse et al., 1999; Lindbäck et al., 2004). The 554 bp full-length sequence showed the highest activity of all P*nhe* constructs, while the 406 bp P*nhe-s1* (P*nhe* lacking PlcR binding site 1) showed a strongly decreased activity (**Figures 2A,B**). This strongly indicates the activating role of PlcR binding site 1, as confirmed by an 11-fold decrease of promoter activity (**Figure 2B**). Thus, the highly conserved PlcR 1 site is responsible for PlcR-dependent *nhe* expression, while the less conserved PlcR 2 site contains a 2 bp central insertion (**Figure 1C**). Deletion of the region containing the upstream-located promoter abolished transcription (construct P*nhe-s2*, **Figures 2A,B**), indicating that this sequence is the active and essential promoter in *B. cereus* INRA C3. The luminescence intensity of a promoter fusion lacking both PlcR binding sites (P*nhe-s3*) was similar to the intensity of the P*nhe-s2* construct, showing that the PlcR binding site 2 does not have an activating function by itself (**Figure 2B**). A promoter fusion lacking the second putative promoter (P*nhe-*Δ5′ UTR) resulted in an activity similar to the control plasmid pXen1 without any active promoter. This demonstrates that this fragment of the promoter region also contains one or more essential activating regulatory elements, which might include a ResD, cre, Fnr, PlcR 2 and a putative SinR binding site. In summary, transcription of the *nhe* operon strongly depends on the presence of the entire IGR, which underpins the necessity of a concerted interaction of all regulatory elements to regulate *nhe* expression.

### *Hbl* 5′ UTR Represses *hbl* Transcription

**FIGURE 3:**
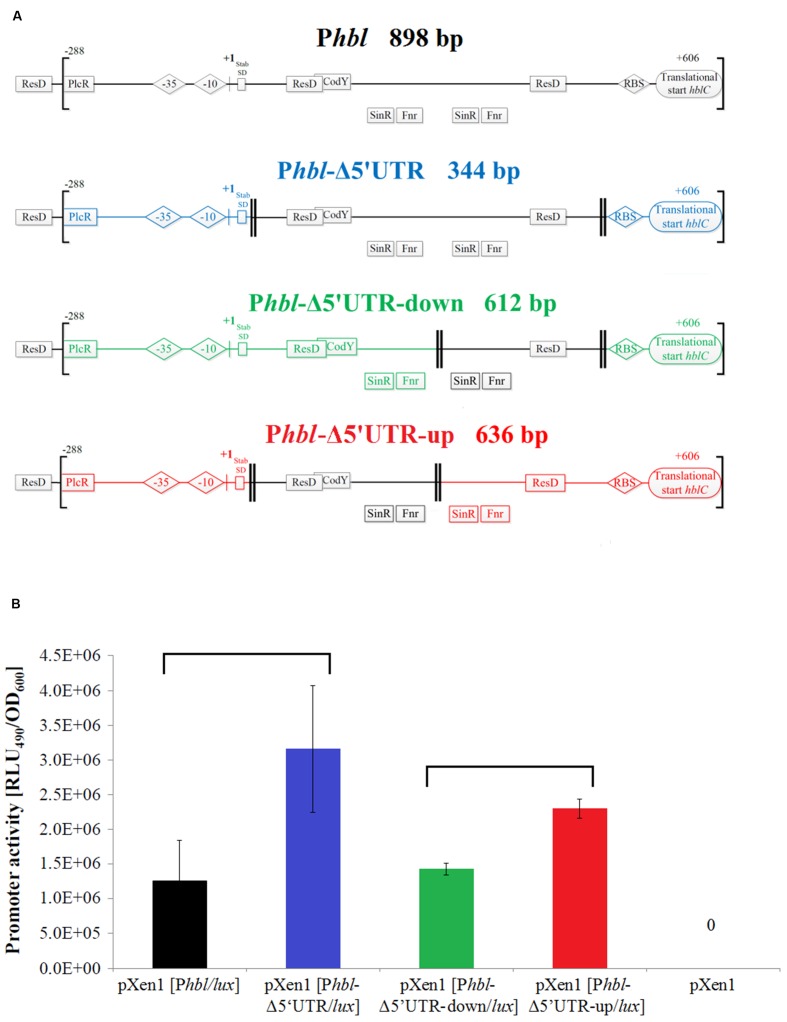
**Promoter activity of complete and partial *hbl* 5′ UTR in *B. cereus* INRA C3. (A)** P*hbl* wild type construct and deletion variants. Regions analyzed in promoter fusions are named and indicated by brackets, double lines enclose deletions and +1 is the TSS determined by 5′ RACE. Promoter elements and (putative) binding sites of transcriptional regulators (compare **Figure 1**) are displayed. **(B)** P*hbl* promoter activities were determined in modified MOD minimal medium in triplicates and compared at the time of peak activity of the construct containing the entire promoter region. Detailed growth and transcription kinetics of *B. cereus* INRA C3 P*hbl* promoter fusions are shown in Supplementary Figure S2B. Luminescence signals generated by the transcription of *lux* genes indicate promoter activity of the 5′ UTR variant tested. Statistically significant differences in transcriptional activity are marked by brackets (*p* < 0.05).

To study the function of the *hbl* 5′ UTR *lux* reporter fusions including partial or complete deletion of the 606 bp 5′ UTR were constructed and transferred into *B. cereus* INRA C3 (**Figure 3A**). The full-length construct contains the highly conserved PlcR binding site 1 (**Figures 1D** and **3A**). Transcription of the *hbl* operon was previously shown to be PlcR-dependent (Agaisse et al., 1999). Interestingly, deletion of the entire 5′ UTR (P*hbl*-Δ5′ UTR) led to an increased promoter activity compared to the full-length construct (**Figure 3B**). In contrast, deletion of the downstream half of the 5′ UTR (P*hbl*-Δ5′ UTR-down) resulted in luminescence levels comparable to the wild type situation. Therefore, the putative ResD, SinR, and Fnr binding sites do not influence promoter activity under our experimental conditions. Deletion of the upstream half of the 5′ UTR (P*hbl*-Δ5′ UTR-up) containing putative binding sites for CodY, ResD, SinR, and Fnr led to a stimulation of transcription, which was less pronounced compared to the deletion of the entire 5′ UTR. We conclude that the 268 bp region designated 5′ UTR-up (**Figure 3A**) partially represses *hbl* transcription in *B. cereus* INRA C3. It has been shown previously that the deletion of the two-component system *yvfTU* leads to a reduced *plcR, papR, nhe, nprB* and *plcB* transcription but an increased *hbl* transcription (Brillard et al., 2008). This confirms not just differences in the regulation of *nhe* and *hbl* expression despite regulation by the same factors, but also an additional PlcR-independent regulation of *hbl* transcription.

### Nutrient Deficiency Activates Transcription of Tripartite Enterotoxins

**FIGURE 4:**
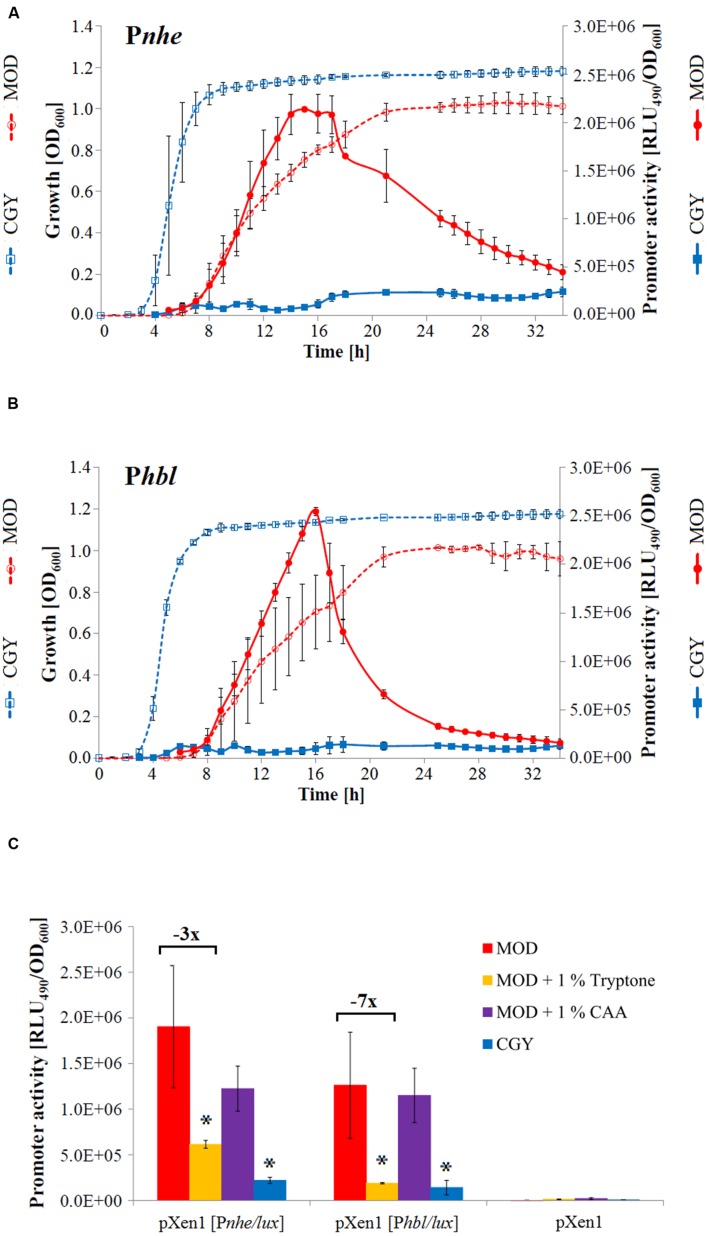
**Activity of the full-length enterotoxin promoter regions in *B. cereus* INRA C3 in media with differing amino acid availability.** Growth and promoter activity kinetics of *B. cereus* INRA C3 pXen1 [P*nhe/lux*] **(A)** and *B. cereus* INRA C3 pXen1 [P*hbl/lux*] **(B)** in complex CGY and defined MOD medium. Cell density was measured at an OD of 600 nm and bioluminescence intensity was recorded for 0.1 s at 490 nm with a luminescence microplate reader. **(C)** Maximal enterotoxin promoter activities of *B. cereus* INRA C3 in different media. Promoter activities were determined in triplicates and peak activities were compared. Luminescence signals generated by an active transcription of the *lux* genes are proportional to the activity of the promoter region tested. Maximum promoter activity of P*nhe* was detected as follows (hours after inoculation): MOD: 17 h, MOD + 1% tryptone: 14 h, MOD + 1% CAA: 15 h, CGY: 18 h. Maximum promoter activity of P*hbl* was detected as follows (hours after inoculation): MOD: 16 h, MOD + 1% tryptone: 13 h, MOD + 1% CAA: 13 h, CGY: 18 h. The increase of promoter activity in MOD + 1% tryptone is shown in relation to unsupplemented MOD medium. Statistically significant differences in transcriptional activity (in comparison to MOD minimal medium) are marked by an asterisk (*p* < 0.05).

Bioluminescent reporter strains containing the wild type 5′ IGRs of *nhe* or *hbl* (P*nhe* and P*hbl*, **Figures 2A** and **3A**) were used to compare the impact of a minimal medium (MOD) and a rich medium (CGY) on enterotoxin expression (Supplementary Table S1). While growth of *B. cereus* INRA C3 was delayed and reduced in the minimal medium, we noticed a steep increase in *nhe* and *hbl* promoter activity in comparison to growth in nutrient and amino acid rich media such as CGY (**Figures 4A,B**). In CGY the maximal promoter activity was reached during stationary phase, while peak activity was detected in the late exponential phase in defined MOD medium.

To further analyze the impact of the availability and accessibility of nitrogen sources, maximal promoter activities were compared in defined MOD medium supplemented with either tryptone or CAAs. Tryptone represents enzymatically digested casein and is a mixture of differently sized oligopeptides (Wang et al., 2013), which are less accessible and need to be degraded by (exo-)proteases, but are thus longer available. In contrast, CAAs are free amino acids obtained by acid hydrolyzation of casein (Nolan, 1971). They are fast and easily taken up and metabolized. In MOD medium supplemented with 1% CAA, *nhe* and *hbl* enterotoxin promoter activity may show a trend to be slightly lower than in unsupplemented MOD (**Figure 4C**). Free amino acids are also present in MOD, albeit in lower amounts, confirming that the depletion of free amino acids is a determining factor for enhanced enterotoxin promoter activity. When *B. cereus* INRA C3 was grown in MOD medium supplemented with 1% tryptone, promoter activities of P*nhe* and P*hbl* were threefold and sevenfold lower than in MOD medium, respectively (**Figure 4C**). These results point to an activation of enterotoxin promoter activity during unfavorable conditions, such as the absence of easily metabolizable amino acids. BCAAs and GTP activate the nutrient-sensitive repressor CodY (Ratnayake-Lecamwasam et al., 2001; Shivers and Sonenshein, 2004; Brinsmade et al., 2010). Thus, we hypothesized that CodY-dependent promoter repression is prolonged in MOD + 1% tryptone, which continuously contains free amino acids from the bacterial degradation of oligopeptides. Detailed growth and transcription kinetics of *B. cereus* INRA C3 in media with different amino acid availability are shown in Supplementary Figure S3. The quorum sensing virulence regulator system PlcR-PapR is known to be indirectly positively controlled by CodY via enhancing the import of the signaling peptide PapR (Slamti et al., 2015) and the enterotoxin genes are under positive transcriptional control of PlcR (Gohar et al., 2008; Ramarao and Sanchis, 2013). In contrast to previous studies, which found the PlcR regulon to be activated by CodY in an unknown manner in an emetic *B. cereus*, an enterotoxic *B. cereus* and a *B. thuringiensis* strain (Frenzel et al., 2012; Lindbäck et al., 2012; Slamti et al., 2015), our results suggest a repression of enterotoxin gene transcription by CodY. *In silico* comparison of the CodY binding consensus sequence with the *nhe* and *hbl* promoter regions revealed several potential binding sites downstream of the promoters. This could result in premature termination of transcription by a roadblock mechanism, as described for the *B. subtilis ybgE* gene (Belitsky and Sonenshein, 2011b). Supplementary Figures S4 and S5 display alignments of the *nhe* and *hbl* promoter regions in *B. cereus*, *B. thuringiensis* and *B. cytotoxicus* strains. The first and second CodY binding sites in P*nhe* are almost identical (Supplementary Figure S4). However, the second binding site in *B. cytotoxicus* CVUAS 2833 is different and located 20 bp farther downstream, which might explain the varying binding affinity. A potential CodY binding site was located in P*nhe* of *B. cereus* F4810/72 (gray underlined), but no binding of CodY to P*nhe* F4810/72 could be detected (Frenzel et al., 2012). This site is not present in P*nhe* CVUAS 2833. P*nhe* INRA C3 and P*nhe* CVUAS 2833 were positive in electrophoretic mobility shift experiments. A possible direct regulation of *hbl* expression by CodY has not yet been analyzed. The *hbl* promoter regions and potential CodY binding sites are highly conserved (Supplementary Figure S5).

### CodY binds to P*hbl* and P*nhe* In vitro

Since the promoter activity studies indicated a strong activation of enterotoxin transcription after depletion of free amino acids, the affinity of CodY to enterotoxin promoter regions was analyzed in the highly enterotoxinogenic *B. cereus* INRA C3 and *B. cytotoxicus* CVUAS 2833 (Guinebretiere et al., 2013; Jeßberger et al., 2015).

**FIGURE 5:**
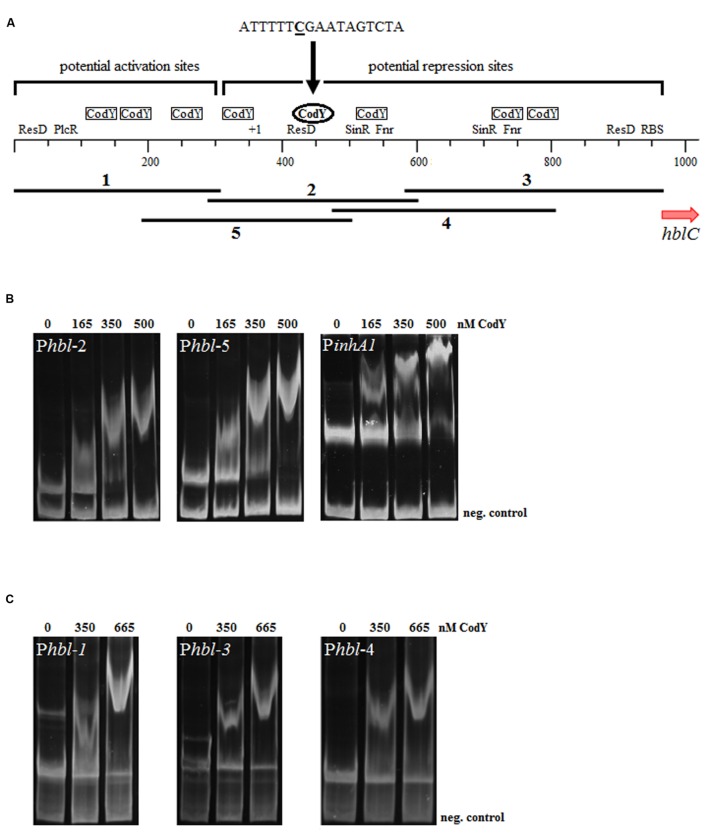
**Determination of CodY affinity to the *hbl* 5′ IGR by gel mobility shift analysis. (A)** The sequences of the *B. cereus* INRA C3 *hbl* 5′ IGR (966 bp) fragments used in electrophoretic mobility shift assays (1–5) can be found in Supplementary Figure S5. All potential CodY binding sites found by an *in silico* analysis are indicated. Sites that contain two or more mismatches to the consensus sequence (Belitsky and Sonenshein, 2013) are boxed. The CodY binding site indicated by an ellipse contains only one mismatch to the consensus sequence. Putative binding sites that would cause a roadblock of transcription are marked as potential repression sites. **(B,C)** Gel mobility shift assays of CodY binding to the *hbl* 5′ IGRs. Reactions contained 100 ng DNA (401–501 fmol) and CodY concentrations are indicated with respect to the monomer. Negative control: 241 bp fragment amplified from the 16S rRNA gene *rrn*. Fragments P*hbl*-2, P*hbl*-5 and positive control P*inhA1* **(B)** are bound by CodY with a high affinity (K_D_ ~250 nM), while fragments P*hbl*-1, P*hbl*-3, and P*hbl*-4 **(C)** bind CodY with a low affinity (K_D_ ~700 nM).

Due to its size of over 600 bp P*hbl* was divided in five fragments each tested in electrophoretic mobility shift assays (**Figure 5A**). Comparison with the consensus sequence (Belitsky and Sonenshein, 2013) identified putative binding sites with more than one mismatch to the consensus sequence in all tested sequences (**Figure 5A** and Supplementary Figure S5). *In vitro* DNA affinity tests revealed that CodY shows a low affinity to the fragments P*hbl*-1, -3, and -4 with an estimated dissociation constant (K_D_) of around 700 nM. One potential repressor binding site with only one mismatch to the consensus sequence was found in P*hbl*-2 and P*hbl*-5 downstream of the promoter (**Figures 1** and **5A**). P*hbl*-2 and P*hbl*-5 were both bound by CodY (**Figure 5B**) with a K_D_ of less than 250 nM. A gel retardation control experiment with the CodY-interacting *inhA1* promoter region (Frenzel et al., 2012) also resulted in a K_D_ of around 250 nM. It is thus likely that CodY binds to P*hbl* at a conserved binding site 84 bp downstream of the TSS (indicated by an ellipse in **Figure 5A**).

In addition, CodY binds to a stretch of the *nhe* 5′ IGR of *B. cereus* INRA C3 (568 bp) and of *B. cytotoxicus* CVUAS 2833 (517 bp) at potential repressor binding positions with a K_D_ of ~125 and ~330 nM, respectively (**Figure 2A**, data not shown). We additionally analyzed CodY affinity to the *cytK* promoter regions, but could neither detect specific interactions with P*cytK-1* nor with P*cytK-2* (K_D_ values > 1000 nM, data not shown).

### Fine-Tuning of Enterotoxin Expression Through Variation of CodY Binding Sites?

In contrast to *B. cereus* INRA C3 (our data), the *B. cereus* F4810/72 *nhe* 5′ IGR is not bound by CodY *in vitro* (Frenzel et al., 2012). These results indicate that CodY has a different affinity to the three putative binding sites in the 5′ IGRs (Supplementary Figure S4).

One site is identical in all three strains, while differences in the second site might cause strain-specific deviation of binding affinity. Both sites are indicated in **Figure 1C**. DNA fragments containing only one of the two sites were negative in gel retardation experiments (data not shown). The third potential binding site (Frenzel et al., 2012) occurs in *B. cereus*, but not in *B. cytotoxicus* leading to the conclusion that it plays a marginal role in CodY-mediated repression of P*nhe* activity. Thus, more than one target site might be necessary to allow effective binding of CodY. The electrophoretic mobility shift assays showed that CodY binds in addition to the main binding site with low affinity to several sites within the *hbl* 5′ IGR (**Figure 5**). These might influence CodY binding *in vivo*. Our results fit to the previously stated hypothesis that the difference in CodY binding strength is one of the determinant factors of the hierarchical CodY regulon expression (Brinsmade et al., 2014). Hierarchical and DNA sequence-dependent binding of several molecules of a single transcriptional regulator was previously also shown for *E. coli* (Yoshida et al., 2006) and might similarly occur in CodY-binding to the enterotoxin 5′ IGRs.

**FIGURE 6:**
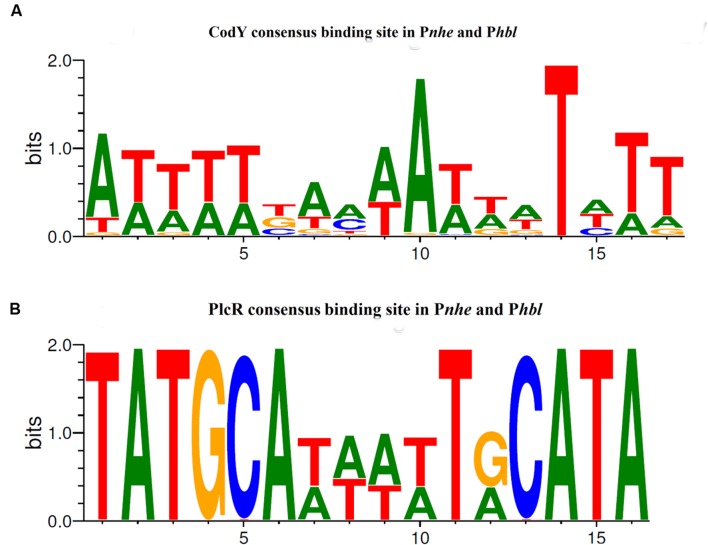
**CodY and PlcR consensus sequences in the promoter regions of *B. cereus sensu lato* enterotoxin encoding genes.** Conservation of the consensus sequences is depicted as logo based on the sequence comparison of P*nhe* and P*hbl* of 142 *B. cereus sensu lato* strains. Strain list and detailed cluster affiliation were described previously (Böhm et al., 2015). **(A)** CodY consensus sequence of three CodY binding sites in 142 strains (based on 379 sequences: one potential site in 142 P*nhe*, one potential site in 140 P*nhe* (not present in the two cluster I *B. pseudomycoides* strains) and one site in 97 P*hbl*). This consensus sequence is highly similar to the CodY consensus binding sequence in *B. subtilis* (Belitsky and Sonenshein, 2013). **(B)** PlcR consensus binding sequence found in all 142 P*nhe* and P*hbl* sequences (based on 239 sequences: 142 P*nhe* (PlcR 1), 97 P*hbl*).

The two putative CodY binding sites in P*nhe* and the confirmed site in P*hbl* were found in almost all of the 142 investigated *B. cereus sensu lato* strains (**Figure 6A**). They show maximally one nucleotide mismatch to the *B. subtilis* consensus sequence (Belitsky and Sonenshein, 2013), but a comparison reveals considerable variability with only one completely conserved T at position 14 and an almost conserved A at position 10. Interestingly, variations at almost all positions within the binding motif have previously been shown to modulate the stringency of CodY binding (Belitsky and Sonenshein, 2011a). Our *in vitro* experiments indicate that a high affinity to the 5′ IGR of *nhe* depends on the sequence of the second CodY binding site (**Figure 1** and Supplementary Figure S4). The C-terminal DNA-binding domain of CodY as well as the dimerizing N-terminal co-factor binding domain (Levdikov et al., 2006) is highly conserved in *B. cereus* group strains (Supplementary Figure S6). The strain-specific differences in CodY affinity and the general lack of CodY binding site conservation support the hypothesis that the binding site sequence, or the combination of different motifs, is crucial for fine-tuning of enterotoxin gene transcription.

In contrast, PlcR binding sites within the enterotoxin promoters reveal a much higher degree of conservation (**Figure 6B**). The sequence of the DNA-binding N-terminal domain of PlcR is conserved, while the regulatory C-terminal regions are variable to allow adaptation to changing conditions (Declerck et al., 2007; Böhm et al., 2015). Therefore, PlcR-mediated activation of enterotoxin transcription is controlled by protein activity and environmental factors, while CodY-mediated repression additionally may depend on strain-specific 5′ UTR sequences.

Being the major virulence regulator in *B. cereus*, PlcR is responsible for the general activation of enterotoxin transcription under unfavorable conditions (Gohar et al., 2008). In contrast, fine-tuning of enterotoxin transcription and, thus, a differential toxin gene regulation might be mediated by CodY binding in a strain-specific manner via affinity to variable CodY binding motifs. Transcription of the construct P*hbl*-Δ5′ UTR still showed media-dependency (Supplementary Figure S7), indicating that not only regulators binding within the 5′ UTR play a role to fine-tune transcription. All putative ResD, Fnr, CodY, cre and PlcR 2 sites are also variable, perhaps indicating that oxygen and other nutrient levels may affect enterotoxin transcription differently in each *B. cereus* strain.

## Conclusion

We present evidence for a high CodY-mediated *nhe* and *hbl* promoter activity under nutrient, especially amino acid limited conditions. While PlcR is the main virulence activator in *B. cereus sensu lato*, CodY may be used for a strain-specific fine-tuning of enterotoxin transcription via repression in response to specific environmental conditions. The unusually long promoter regions of *nhe* and *hbl* might be important for a concomitant interaction of several global regulators. The redox regulators ResD and Fnr, for instance, interact not only directly with their DNA recognition sites, but are also capable of interaction with each other (Esbelin et al., 2009) and formation of a ternary complex with the virulence regulator PlcR (Esbelin et al., 2012).

However, the actual enterotoxin synthesis in *B. cereus* is rarely consistent with transcriptional activity and is, moreover, highly strain-specific (Ceuppens et al., 2013; Jeßberger et al., 2015). It might be speculated that the 5′ UTRs, in addition, interfere with post-transcriptional and/or translational processes (Esbelin et al., 2008) to modify the efficiency of enterotoxin production according to the environmental conditions prevalent in the human intestine.

## Author Contributions

M-EB performed the experiments. VK helped with the construction of mutants and the *lux* reporter experiments, and also with the design of the study. NJ was involved in compiling up the strain collection and critically revised the work. EF helped with the EMSA assays and critically revised the paper. SS was involved in the design of the study and wrote the paper together with M-EB. All authors worked on the manuscript. All authors finally approved the version to be published and agree to be accountable for all aspects of the work in ensuring that questions related to the accuracy or integrity of any part of the work are appropriately investigated and resolved.

## Footnotes

1 https://www.ebi.ac.uk/Tools/msa/clustalo/

2 http://weblogo.threeplusone.com/create.cgi

3 http://www.bioinformatics.org/sms2/orf_find.html

4 https://www.R-project.org

## Abbreviations

BCAA: branched-chain amino acid
CAAs: casamino acids
5′ IGR: 5′ intergenic region
5′ UTR: 5′ untranslated region
TSS: transcription start site.

## References

[B1] Agaisse H. Gominet M. Okstad O. A. Kolsto A. B. Lereclus D. (1999). PlcR is a pleiotropic regulator of extracellular virulence factor gene expression in *Bacillus thuringiensis*. *Mol. Microbiol.* 32 1043–1053. 10.1046/j.1365-2958.1999.01419.x 10361306

[B2] Agaisse H. Lereclus D. (1996). STAB-SD: a shine-dalgarno sequence in the 5’ untranslated region is a determinant of mRNA stability. *Mol. Microbiol.* 20 633–643. 10.1046/j.1365-2958.1996.5401046.x 8736542

[B3] Ash C. Farrow J. A. Dorsch M. Stackebrandt E. Collins M. D. (1991). Comparative analysis of *Bacillus anthracis*, *Bacillus cereus*, and related species on the basis of reverse transcriptase sequencing of 16S rRNA. *Int. J. Syst. Bacteriol.* 41 343–346. 1715736 10.1099/00207713-41-3-343

[B4] Barrie D. Wilson J. A. Hoffman P. N. Kramer J. M. (1992). *Bacillus cereus* meningitis in two neurosurgical patients: an investigation into the source of the organism. *J. Infect.* 25 291–297. 10.1016/0163-4453(92)91579-Z 1474265

[B5] Beecher D. J. Wong A. C. (1994). Improved purification and characterization of hemolysin BL, a hemolytic dermonecrotic vascular permeability factor from *Bacillus cereus*. *Infect. Immun.* 62 980–986. 8112873 10.1128/iai.62.3.980-986.1994 PMC186213

[B6] Belitsky B. R. Sonenshein A. L. (2011a). Contributions of multiple binding sites and effector-independent binding to CodY-mediated regulation in *Bacillus subtilis*. *J. Bacteriol.* 193 473–484. 10.1128/JB.01151-10 21097623 PMC3019828

[B7] Belitsky B. R. Sonenshein A. L. (2011b). Roadblock repression of transcription by *Bacillus subtilis* CodY. *J. Mol. Biol.* 411 729–743. 10.1016/j.jmb.2011.06.012 21699902 PMC3156270

[B8] Belitsky B. R. Sonenshein A. L. (2013). Genome-wide identification of *Bacillus subtilis* CodY-binding sites at single-nucleotide resolution. *Proc. Natl. Acad. Sci. U.S.A.* 110 7026–7031. 10.1073/pnas.1300428110 23569278 PMC3637721

[B9] Böhm M. E. Huptas C. Krey V. M. Scherer S. (2015). Massive horizontal gene transfer, strictly vertical inheritance and ancient duplications differentially shape the evolution of *Bacillus cereus* enterotoxin operons hbl, cytK and nhe. *BMC Evol. Biol.* 15:246 10.1186/s12862-015-0529-4 PMC4641410 26555390

[B10] Brillard J. Lereclus D. (2004). Comparison of cytotoxin cytK promoters from *Bacillus cereus* strain ATCC 14579 and from a *B. cereus* food-poisoning strain. *Microbiology* 150 2699–2705. 10.1099/mic.0.27069-0 15289566

[B11] Brillard J. Susanna K. Michaud C. Dargaignaratz C. Gohar M. Nielsen-Leroux C. (2008). The YvfTU two-component system is involved in plcR expression in *Bacillus cereus*. *BMC Microbiol.* 8:183 10.1186/1471-2180-8-183 PMC2588459 18925929

[B12] Brinsmade S. R. Alexander E. L. Livny J. Stettner A. I. Segre D. Rhee K. Y. (2014). Hierarchical expression of genes controlled by the *Bacillus subtilis* global regulatory protein CodY. *Proc. Natl. Acad. Sci. U.S.A.* 111 8227–8232. 10.1073/pnas.1321308111 24843172 PMC4050614

[B13] Brinsmade S. R. Kleijn R. J. Sauer U. Sonenshein A. L. (2010). Regulation of CodY activity through modulation of intracellular branched-chain amino acid pools. *J. Bacteriol.* 192 6357–6368. 10.1128/JB.00937-10 20935095 PMC3008522

[B14] Ceuppens S. Boon N. Uyttendaele M. (2013). Diversity of *Bacillus cereus* group strains is reflected in their broad range of pathogenicity and diverse ecological lifestyles. *FEMS Microbiol. Ecol.* 84 433–450. 10.1111/1574-6941.12110 23488744

[B15] Chu F. Kearns D. B. Branda S. S. Kolter R. Losick R. (2006). Targets of the master regulator of biofilm formation in *Bacillus subtilis*. *Mol. Microbiol.* 59 1216–1228. 10.1111/j.1365-2958.2005.05019.x 16430695

[B16] Daelman J. Membre J. M. Jacxsens L. Vermeulen A. Devlieghere F. Uyttendaele M. (2013). A quantitative microbiological exposure assessment model for *Bacillus cereus* in REPFEDs. *Int. J. Food Microbiol.* 166 433–449. 10.1016/j.ijfoodmicro.2013.08.004 24029028

[B17] Damgaard P. H. Granum P. E. Bresciani J. Torregrossa M. V. Eilenberg J. Valentino L. (1997). Characterization of *Bacillus thuringiensis* isolated from infections in burn wounds. *FEMS Immunol. Med. Microbiol.* 18 47–53. 10.1111/j.1574-695X.1997.tb01026.x 9215586

[B18] David D. B. Kirkby G. R. Noble B. A. (1994). *Bacillus cereus* endophthalmitis. *Br. J. Ophthalmol.* 78 577–580. 10.1136/bjo.78.7.577 7918273 PMC504868

[B19] Declerck N. Bouillaut L. Chaix D. Rugani N. Slamti L. Hoh F. (2007). Structure of PlcR: insights into virulence regulation and evolution of quorum sensing in Gram-positive bacteria. *Proc. Natl. Acad. Sci. U.S.A.* 104 18490–18495. 10.1073/pnas.0704501104 17998541 PMC2141804

[B20] den Hengst C. D. Curley P. Larsen R. Buist G. Nauta A. van Sinderen D. (2005). Probing direct interactions between CodY and the oppD promoter of *Lactococcus lactis*. *J. Bacteriol.* 187 512–521. 10.1128/JB.187.2.512-521.2005 15629923 PMC543541

[B21] Dietrich R. Moravek M. Burk C. Granum P. E. Märtlbauer E. (2005). Production and characterization of antibodies against each of the three subunits of the *Bacillus cereus* nonhemolytic enterotoxin complex. *Appl. Environ. Microbiol.* 71 8214–8220. 10.1128/AEM.71.12.8214-8220.2005 16332805 PMC1317347

[B22] Dommel M. K. Frenzel E. Strasser B. Blochinger C. Scherer S. Ehling-Schulz M. (2010). Identification of the main promoter directing cereulide biosynthesis in emetic *Bacillus cereus* and its application for real-time monitoring of ces gene expression in foods. *Appl. Environ. Microbiol.* 76 1232–1240. 10.1128/AEM.02317-09 20038713 PMC2820966

[B23] Ehling-Schulz M. Fricker M. Scherer S. (2004). *Bacillus cereus*, the causative agent of an emetic type of food-borne illness. *Mol. Nutr. Food Res.* 48 479–487. 10.1002/mnfr.200400055 15538709

[B24] Ehling-Schulz M. Vukov N. Schulz A. Shaheen R. Andersson M. Martlbauer E. (2005). Identification and partial characterization of the nonribosomal peptide synthetase gene responsible for cereulide production in emetic *Bacillus cereus*. *Appl. Environ. Microbiol.* 71 105–113. 10.1128/AEM.71.1.105-113.2005 15640177 PMC544239

[B25] Esbelin J. Armengaud J. Zigha A. Duport C. (2009). ResDE-dependent regulation of enterotoxin gene expression in *Bacillus cereus*: evidence for multiple modes of binding for ResD and interaction with Fnr. *J. Bacteriol.* 191 4419–4426. 10.1128/JB.00321-09 19395489 PMC2698483

[B26] Esbelin J. Jouanneau Y. Armengaud J. Duport C. (2008). ApoFnr binds as a monomer to promoters regulating the expression of enterotoxin genes of *Bacillus cereus*. *J. Bacteriol.* 190 4242–4251. 10.1128/JB.00336-08 18424517 PMC2446755

[B27] Esbelin J. Jouanneau Y. Duport C. (2012). *Bacillus cereus* Fnr binds a [4Fe-4S] cluster and forms a ternary complex with ResD and PlcR. *BMC Microbiol.* 12:125 10.1186/1471-2180-12-125 PMC3520743 22731107

[B28] Fagerlund A. Dubois T. Okstad O. A. Verplaetse E. Gilois N. Bennaceur I. (2014). SinR controls enterotoxin expression in *Bacillus thuringiensis* biofilms. *PLoS ONE* 9:e87532 10.1371/journal.pone.0087532 PMC3909190 24498128

[B29] Fagerlund A. Lindbäck T. Storset A. K. Granum P. E. Hardy S. P. (2008). *Bacillus cereus* Nhe is a pore-forming toxin with structural and functional properties similar to the ClyA (HlyE, SheA) family of haemolysins, able to induce osmotic lysis in epithelia. *Microbiology* 154 693–704. 10.1099/mic.0.2007/014134-0 18310016

[B30] Francis K. P. Yu J. Bellinger-Kawahara C. Joh D. Hawkinson M. J. Xiao G. (2001). Visualizing pneumococcal infections in the lungs of live mice using bioluminescent *Streptococcus pneumoniae* transformed with a novel gram-positive lux transposon. *Infect. Immun.* 69 3350–3358. 10.1128/IAI.69.5.3350-3358.2001 11292758 PMC98294

[B31] Frankland G. C. Frankland P. F. (1887). Studies on some new micro-organisms obtained from air. *Philos. Trans. R. Soc. B Biol. Sci.* 178 257–287. 10.1098/rstb.1887.0011

[B32] Frenzel E. Doll V. Pauthner M. Lucking G. Scherer S. Ehling-Schulz M. (2012). CodY orchestrates the expression of virulence determinants in emetic *Bacillus cereus* by impacting key regulatory circuits. *Mol. Microbiol.* 85 67–88. 10.1111/j.1365-2958.2012.08090.x 22571587

[B33] Geng H. Zhu Y. Mullen K. Zuber C. S. Nakano M. M. (2007). Characterization of ResDE-dependent fnr transcription in *Bacillus subtilis*. *J. Bacteriol.* 189 1745–1755. 10.1128/JB.01502-06 17189364 PMC1855754

[B34] Gohar M. Faegri K. Perchat S. Ravnum S. Okstad O. A. Gominet M. (2008). The PlcR virulence regulon of *Bacillus cereus*. *PLoS ONE* 3:e2793 10.1371/journal.pone.0002793 PMC2464732 18665214

[B35] Guinebretiere M. H. Auger S. Galleron N. Contzen M. De Sarrau B. De Buyser M. L. (2013). *Bacillus cytotoxicus* sp. nov. is a novel thermotolerant species of the *Bacillus cereus* group occasionally associated with food poisoning. *Int. J. Syst. Evol. Microbiol.* 63 31–40. 10.1099/ijs.0.030627-0 22328607

[B36] Guinebretiere M. H. Broussolle V. Nguyen-The C. (2002). Enterotoxigenic profiles of food-poisoning and food-borne *Bacillus cereus* strains. *J. Clin. Microbiol.* 40 3053–3056. 10.1128/JCM.40.8.3053-3056.2002 12149378 PMC120679

[B37] Guinebretiere M. H. Thompson F. L. Sorokin A. Normand P. Dawyndt P. Ehling-Schulz M. (2008). Ecological diversification in the *Bacillus cereus* Group. *Environ. Microbiol.* 10 851–865. 10.1111/j.1462-2920.2007.01495.x 18036180

[B38] Handke L. D. Shivers R. P. Sonenshein A. L. (2008). Interaction of *Bacillus subtilis* CodY with GTP. *J. Bacteriol.* 190 798–806. 10.1128/JB.01115-07 17993518 PMC2223590

[B39] Helgason E. Caugant D. A. Olsen I. Kolsto A. B. (2000). Genetic structure of population of *Bacillus cereus* and *B. thuringiensis* isolates associated with periodontitis and other human infections. *J. Clin. Microbiol.* 38 1615–1622. 10747152 10.1128/jcm.38.4.1615-1622.2000 PMC86502

[B40] Hong H. A. Duc Le H. Cutting S. M. (2005). The use of bacterial spore formers as probiotics. *FEMS Microbiol. Rev.* 29 813–835. 10.1016/j.femsre.2004.12.001 16102604

[B41] Jeßberger N. Krey V. M. Rademacher C. Böhm M.-E. Mohr A.-K. Ehling Schulz M. (2015). From genome to toxicity: a combinatory approach highlights the complexity of enterotoxin production in *Bacillus cereus*. *Front. Microbiol.* 6:560 10.3389/fmicb.2015.00560 PMC4462024 26113843

[B42] Johansson J. Mandin P. Renzoni A. Chiaruttini C. Springer M. Cossart P. (2002). An RNA thermosensor controls expression of virulence genes in *Listeria monocytogenes*. *Cell* 110 551–561. 10.1016/S0092-8674(02)00905-4 12230973

[B43] Kearns D. B. Chu F. Branda S. S. Kolter R. Losick R. (2005). A master regulator for biofilm formation by *Bacillus subtilis*. *Mol. Microbiol.* 55 739–749. 10.1111/j.1365-2958.2004.04440.x 15661000

[B44] Kristoffersen S. M. Tourasse N. J. Kolsto A. B. Okstad O. A. (2011). Interspersed DNA repeats bcr1-bcr18 of *Bacillus cereus* group bacteria form three distinct groups with different evolutionary and functional patterns. *Mol. Biol. Evol.* 28 963–983. 10.1093/molbev/msq269 20961964

[B45] Kuroki R. Kawakami K. Qin L. Kaji C. Watanabe K. Kimura Y. (2009). Nosocomial bacteremia caused by biofilm-forming *Bacillus cereus* and *Bacillus thuringiensis*. *Intern. Med.* 48 791–796. 10.2169/internalmedicine.48.1885 19443973

[B46] Levdikov V. M. Blagova E. Joseph P. Sonenshein A. L. Wilkinson A. J. (2006). The structure of CodY, a GTP- and isoleucine-responsive regulator of stationary phase and virulence in gram-positive bacteria. *J. Biol. Chem.* 281 11366–11373. 10.1074/jbc.M513015200 16488888

[B47] Lindbäck T. Fagerlund A. Rodland M. S. Granum P. E. (2004). Characterization of the *Bacillus cereus* Nhe enterotoxin. *Microbiology* 150 3959–3967. 10.1099/mic.0.27359-0 15583149

[B48] Lindbäck T. Mols M. Basset C. Granum P. E. Kuipers O. P. Kovacs A. T. (2012). CodY, a pleiotropic regulator, influences multicellular behaviour and efficient production of virulence factors in *Bacillus cereus*. *Environ. Microbiol.* 14 2233–2246. 10.1111/j.1462-2920.2012.02766.x 22540344

[B49] Lindbäck T. Okstad O. A. Rishovd A. L. Kolsto A. B. (1999). Insertional inactivation of hblC encoding the L2 component of *Bacillus cereus* ATCC 14579 haemolysin BL strongly reduces enterotoxigenic activity, but not the haemolytic activity against human erythrocytes. *Microbiology* 145(Pt 11), 3139–3146. 10.1099/00221287-145-11-3139 10589721

[B50] Loh E. Gripenland J. Johansson J. (2006). Control of *Listeria monocytogenes* virulence by 5’-untranslated RNA. *Trends Microbiol.* 14 294–298. 10.1016/j.tim.2006.05.001 16730443

[B51] Lund T. De Buyser M. L. Granum P. E. (2000). A new cytotoxin from *Bacillus cereus* that may cause necrotic enteritis. *Mol. Microbiol.* 38 254–261. 10.1046/j.1365-2958.2000.02147.x 11069652

[B52] Majerczyk C. D. Dunman P. M. Luong T. T. Lee C. Y. Sadykov M. R. Somerville G. A. (2010). Direct targets of CodY in *Staphylococcus aureus*. *J. Bacteriol.* 192 2861–2877. 10.1128/JB.00220-10 20363936 PMC2876493

[B53] Narberhaus F. Waldminghaus T. Chowdhury S. (2006). RNA thermometers. *FEMS Microbiol. Rev.* 30 3–16. 10.1111/j.1574-6976.2005.004.x 16438677

[B54] Nawrocki E. P. Burge S. W. Bateman A. Daub J. Eberhardt R. Y. Eddy S. R. (2015). Rfam 12.0: updates to the RNA families database. *Nucleic Acids Res.* 43 D130–D137. 10.1093/nar/gku1063 25392425 PMC4383904

[B55] Neuhaus K. Landstorfer R. Fellner L. Simon S. Schafferhans A. Goldberg T. (2016). Translatomics combined with transcriptomics and proteomics reveals novel functional, recently evolved orphan genes in *Escherichia coli* O157:H7 (EHEC). *BMC Genomics* 17:133 10.1186/s12864-016-2456-1 PMC4765031 26911138

[B56] Nolan R. A. (1971). Amino acids and growth factors in vitamin-free casamino acids. *Mycologia* 63 1231–1234. 10.2307/3757997 5156995

[B57] Ramarao N. Sanchis V. (2013). The pore-forming haemolysins of *Bacillus cereus*: a review. *Toxins (Basel)* 5 1119–1139. 10.3390/toxins5061119 23748204 PMC3717773

[B58] Ratnayake-Lecamwasam M. Serror P. Wong K. W. Sonenshein A. L. (2001). *Bacillus subtilis* CodY represses early-stationary-phase genes by sensing GTP levels. *Genes Dev.* 15 1093–1103. 10.1101/gad.874201 11331605 PMC312684

[B59] Ryan P. Macmillan J. D. Zilinskas B. A. (1997). Molecular cloning and characterization of the genes encoding the L1 and L2 components of hemolysin BL from *Bacillus cereus*. *J. Bacteriol.* 179 5. 10.1128/jb.179.8.2551-2556.1997 PMC179003 9098052

[B60] Shen A. Higgins D. E. (2005). The 5′ untranslated region-mediated enhancement of intracellular listeriolysin O production is required for *Listeria monocytogenes* pathogenicity. *Mol. Microbiol.* 57 1460–1473. 10.1111/j.1365-2958.2005.04780.x 16102013

[B61] Shinagawa K. (1990). Analytical methods for *Bacillus cereus* and other *Bacillus* species. *Int. J. Food Microbiol.* 10 125–141. 10.1016/0168-1605(90)90061-9 2119209

[B62] Shivers R. P. Sonenshein A. L. (2004). Activation of the *Bacillus subtilis* global regulator CodY by direct interaction with branched-chain amino acids. *Mol. Microbiol.* 53 599–611. 10.1111/j.1365-2958.2004.04135.x 15228537

[B63] Slamti L. Lemy C. Henry C. Guillot A. Huillet E. Lereclus D. (2015). CodY regulates the activity of the virulence quorum sensor PlcR by controlling the import of the signaling peptide PapR in *Bacillus thuringiensis*. *Front. Microbiol.* 6:1501 10.3389/fmicb.2015.01501 PMC4701985 26779156

[B64] Sonenshein A. L. (2005). CodY, a global regulator of stationary phase and virulence in Gram-positive bacteria. *Curr. Opin. Microbiol.* 8 203–207. 10.1016/j.mib.2005.01.001 15802253

[B65] Stenfors Arnesen L. P. Fagerlund A. Granum P. E. (2008). From soil to gut: *Bacillus cereus* and its food poisoning toxins. *FEMS Microbiol. Rev.* 32 579–606. 10.1111/j.1574-6976.2008.00112.x 18422617

[B66] van der Voort M. Kuipers O. P. Buist G. De Vos W. M. Abee T. (2008). Assessment of CcpA-mediated catabolite control of gene expression in *Bacillus cereus* ATCC 14579. *BMC Microbiol.* 8:62 10.1186/1471-2180-8-62 PMC2358912 18416820

[B67] Wang J. Su Y. Jia F. Jin H. (2013). Characterization of casein hydrolysates derived from enzymatic hydrolysis. *Chem. Cent. J.* 7 62 10.1186/1752-153X-7-62 PMC3626679 23556455

[B68] Winkler W. C. Breaker R. R. (2005). Regulation of bacterial gene expression by riboswitches. *Annu. Rev. Microbiol.* 59 487–517. 10.1146/annurev.micro.59.030804.121336 16153177

[B69] Wong K. K. Bouwer H. G. Freitag N. E. (2004). Evidence implicating the 5’ untranslated region of *Listeria monocytogenes* actA in the regulation of bacterial actin-based motility. *Cell Microbiol.* 6 155–166. 10.1046/j.1462-5822.2003.00348.x 14706101

[B70] Wray L. V. Jr. Fisher S. H. (2011). *Bacillus subtilis* CodY operators contain overlapping CodY binding sites. *J. Bacteriol.* 193 4841–4848. 10.1128/JB.05258-11 21764931 PMC3165709

[B71] Yoshida T. Qin L. Egger L. A. Inouye M. (2006). Transcription regulation of ompF and ompC by a single transcription factor, OmpR. *J. Biol. Chem.* 281 17114–17123. 10.1074/jbc.M602112200 16618701

[B72] Zuker M. (2003). Mfold web server for nucleic acid folding and hybridization prediction. *Nucleic Acids Res.* 31 3406–3415. 10.1093/nar/gkg595 12824337 PMC169194

