# Supplementary material for: Comparative Bioinformatics and Experimental Analysis of the Intergenic Regulatory Regions of Bacillus cereus hbl and nhe Enterotoxin Operons and the Impact of CodY on Virulence Heterogeneity
Source: Front Microbiol. 2016 May 24;7:768. doi: 10.3389/fmicb.2016.00768 (PMC4877379; doi:10.3389/fmicb.2016.00768)
Supplement: Supplementary file 1 [file Data_Sheet_1.PDF]

Table S1: Composition of media used to compare growth and enterotoxin expression of *B. cereus* INRA C3.

To obtain MOD + 1 % casamino acids (CAA) and MOD + 1 % tryptone, additional 10 g/l CAA or tryptone were dissolved and autoclaved together with the MOD medium components.

**CGY:**	**MOD:**	**MOD trace elements:** (1000x)
20 g/l CAA	6 g/l $(NH_4)_2SO_4$	675 mg/l $FeCl_2$ x 6 H_2O
6 g/l yeast extract	1 g/l K_2HPO_4 (1000x)	50 mg/l $MnCl_2$ x 4 H_2O
2 g/l $(NH_4)_2SO_4$	0.04 g/l $MgSO_4$ x 7 H_2O	30 mg/l Na_2MoO_4 x 2 H_2O
14 g/l K_2HPO_4	2 g/l L-glutamic acid	275 mg/l $CaCl_2$
6 g/l KH_2PO_4	0.39 g/l L-glycine	85 mg/l $ZnCl_2$
1 g/l $Na_3C_6H_5O_7$ x 2 H_2O	0.91 g/l L-valine	30 mg/l $CoCl_2$ x 6 H_2O
2 g/l $MgSO_4$ x 7 H_2O	0.91 g/l L-threonine	40 mg/l $CuSO_4$
100 ml 10 % D-glucose	0.4 g/l L-methionine	24 mg/l $NaSeO_4$
	0.36 g/l L-histidine	
	0.46 g/l L-arginine	
	0.91 g/l L-aspartic acid	
	0.04 g/l L-cysteine	
	0.7 g/l L-isoleucine	
	1.37 g/l L-leucine	
	0.28 g/l L-phenylalanine	
	1.18 g/l L-lysine	
	0.66 g/l L-serine	
	0.042 g/l L-tyrosine	
	10 ml/l 2 M D-glucose	

Table S2: Primer designed in this study.

Restriction sites within primers for the construction of pXen1 promoter fusion plasmids are highlighted in bold. Gene specific primers were used with the Invitrogen 5'RACE system version 2.0.

Primer	Sequence [5´→3´]	Restric-tion site	Product and target
**GSP1_hbl**	TAACTGTACATTAGGAC	-	specific to *hbl* of INRA C3
**GSP2_hbl**	CTGCTTGAACGATTGGTGTTGCGAGAG	-	amplification of *hbl* 5'RACE product
**GSP1_nhe**	CTAATTGAATTGGATAATG	-	specific to *nhe* of INRA C3
**GSP2_nhe**	CTCGTAGATACTGCTGTAACCAATAACCCTG	-	amplification of *nhe* 5'RACE product
**Nhe rev**	AGTC**GGATCC**ACTTTAACTCCTC	BamHI	
**Nhe for & Nhe rev**	ATCC**GAATTC**TGTATATGCTATGC	EcoRI	**pXen1 [P_{nhe}/*lux*]**, 554 bp promoter region
**Nhe-s1 for & Nhe rev**	GGA**GAATTC**AGCTTGAAAATAAAGGG	EcoRI	**pXen1 [$P_{nhe\text{-}s1}$/*lux*]**, 406 bp promoter region
**Nhe-s2 for & Nhe rev**	AAT**GAATTC**ACTGTGTGAATGTGGG	EcoRI	**pXen1 [$P_{nhe\text{-}s2}$/*lux*]**, 247 bp promoter region
**Nhe-s3 for & Nhe rev**	GCA**GAATTC**GTTGGGAGAGATG	EcoRI	**pXen1 [$P_{nhe\text{-}s3}$/*lux*]**, 138 bp promoter region
**Pnhe_A**<br>**Pnhe_B**<br>**Pnhe_C**<br>**Pnhe_D**	AAAATTCATTGAACTATGCG<br>TTT**CCCGGG**AGTTTTGTACTGTAAGGTC<br>AAA**CCCGGG**TAATAAAATGCGATAGTAAT<br>TCATCGGCTTTAATTGATAAG	-<br>XmaI<br>XmaI<br>-	**pXen1 [$P_{nhe\text{-}\Delta5'UTR}$/*lux*]**, 289 bp promoter region
**Hbl for**<br>**Hbl rev**	TATC**GAATTC**TTATGCAATTATAC<br>CTT**GGATCC**GTACACTC	EcoRI<br>BamHI	**pXen1 [P_{hbl}/*lux*]**, 898 bp promoter region
**Phbl_A**<br>**Phbl_B**<br>**Phbl_C**<br>**Phbl_D**	ATATAAGCTTGTTATCCGCTG<br>TAG**CCCGGG**CTTATCCTTTCTGTCTGG<br>AAC**CCCGGG**AAAGGAGTGTACGGAATG<br>CCTGGTATTAATGCAATTTGC	-<br>XmaI<br>XmaI<br>-	**pXen1 [$P_{hbl\text{-}\Delta5'UTR}$/*lux*]**, 344 bp promoter region
**Phbl-s1-B & Phbl_A**	GCT**CCCGGG**TAATCTTGCCATAACATTG	XmaI	**pXen1 [$P_{hbl\text{-}\Delta5'UTR\text{-}down}$/*lux*]**, 612 bp promoter region
**Phbl-s2-C & Phbl_D**	CA**CCCGGG**ACTGTAAGCATTGGTTTATTG	XmaI	**pXen1 [$P_{hbl\text{-}\Delta5'UTR\text{-}up}$/*lux*]**, 636 bp promoter region

**pXen for**	GTTGGGTAACGCCAGGG	-	Different length, 99 bp without insert, multiple cloning site of pXen1
**pXen rev**	CATAGAGAGTCCTCCTCTTG	-	

**CodY-C3-for**	CTTTT**CATATG**GAATTATTAGCAAAAACG	NdeI	*codY*, 814 bp
**CodY-C3-rev**	CTC**CTCGAG**GGAGAGTTTTTTATAAATTA	XhoI	
**CodY-CVUAS-for**	GAACTTTT**CATATG**GAATTATTAGCAAAAAC	NdeI	*codY*, 818 bp
**CodY-CVUAS-rev**	ATT**CTCGAG**GAAAGCTTTTTACTTACATTAG	XhoI	

Table S3: Primer used to amplify promoter fragments for gel mobility shift assays.

Product lengths refer to *B. cereus* INRA C3 or *B. cytotoxicus* CVUAS 2833. All PCR products were diluted to a concentration of 100 ng/µl.

**Primer**	**Product length [bp]**	**Molarity [fmol/µl]**	**Sequence [5´→3´]**	**Target**	**Reference**
	***B. cereus* INRA C3**				
**hbl-1-f**	307	501	AATAATGATATTAGGATGTTTTGTG	P*hbl* of *B. cereus* INRA C3	This study
**hbl-1-r**			GATTACGATCGATAATTTACTG		
**hbl-2-f**	313	492	AAATTATCGATCGTAATCGAC	P*hbl* of *B. cereus* INRA C3	This study
**hbl-2-r**			TCAGCAAACTCCTTACTAG		
**hbl-3-f**	384	401	TCTAGTAAGGAGTTTGCTG	P*hbl* of *B. cereus* INRA C3	This study
**hbl-3-r**			ATTCCGTACACTCCTTTAC		
**hbl-4-f**	334	461	CAAGTTTGTAATAAACGTGTTC	P*hbl* of *B. cereus* INRA C3	This study
**hbl-4-r**			CCTCTCACTTCGATACTC		
**hbl-5-f**	313	492	GTATACACATTAATTTGTAATCATTAC	P*hbl* of *B. cereus* INRA C3	This study
**hbl-5-r**			GAACACGTTTATTACAAACTTG		
**Nhe for**	568	271	ATCCGAATTCTGTATATGCTATGC	P*nhe* of *B. cereus* INRA C3	This study
**Nhe rev**			AGTCGGATCCACTTTAACTCCTC		
**CytK2-f**	330	466	GATAGTGATGTTGCGTTTATTGC	P*cytK-2* of *B. cereus* INRA C3	This study

**CytK2-r**			CAATCACTTCCTTTTATCTTTGTCG		
**inhA1_EMSA_F**	350	440	ATGTAATTCCTCCCTAATTATCGGTC	P*inhA1* of *B. cereus* INRA C3	(Frenzel et al., 2012)
**inhA1_EMSA_R**			TTGTTCATCCCTTATTTCCTCCCCTA		
	B. cytotoxicus **CVUAS 2833**				
**nhe-CVUAS-F**	517	298	AACCGACTTATAGGCAGCC	P*nhe* of *B. cytotoxicus* CVUAS 2833	This study
**nhe-CVUAS-R**			ACTTTAATTCCTCCTAATGTATAAATTAAC		
**CytK1-f**	360	427	TCCTTTACTATTACTATCACCTCTAC	P*cytK-1* of *B. cytotoxicus* CVUAS 2833	This study
**CytK1-r**			CATAGAATCACTTCCTATTTTGTCG		
**inhA1-CVUAS-F**	367	419	AATTTATTCCTCCCTAATTGTCGG	P*inhA1* of *B. cytotoxicus* CVUAS 2833	This study
**inhA1-CVUAS-R**			TTCATCCCCAGTTTTCCTCC		
	**Negative control**				
**16SA1**	241	638	GGAGGAAGGTGGGGATGACG	16S rRNA gene *rrn*	(Martineau et al., 1996)
**16SA2**			ATGGTGTGACGGGCGGTGTG		

Fig. S1: Transcriptional regulator recognition sites in the 5'IGRs of *nhe* and *hbl* in *B. cereus* INRA C3. Binding sites of SinR, CodY and the Stab-SD were determined by *in silico* sequence comparison, the other regulator binding sites were previously experimentally determined in *B. cereus*. Mismatches to the consensus sequences of PlcR, SinR, CodY and Stab-SD are highlighted in bold. Due to low conservation and overlaps, mismatches to the consensus sequences of Fnr, ResD and cre sites are not marked.

> *nhe* 5'IGR *B. cereus* INRA C3

ATCCTTTTTGTGTATATGCTATGCATAATTGCATACGGGGTTAAAAATTATATCAATATA

ResD PlcR 1

ATTTTACCGGTTGTTTGCAAGTAGTGTTTTCTGTTGTGATATGTAGATATTTCAATCGAT

AAAGTGAATCGGATTATATTTTGAAATAAGGACATGAAAGCTTGAAAATAAAGGGAATGA

AAATACTTCTTTACGTTATATAAACAGCTGTGTTTAAATGACCTTACAGTACAAAACTTA

TATGAAAACAAAACGAAATTTCATATTTACGTATGTTGTTTGGTTTAAAATGAATAATAT

CodY

TTT*TATGTAATATATTCACTG*TGTGAATGTGGGAATTTCGATGAAATAGAAAATTTAT**AC**

ResD CodY

AATATT**A**T**T**CATACTAAACACATATGAAAAGTTTTATAACAATAAGTAAGTACTGTAGCA

PlcR 2 (central insertion: N_6 instead of N_4)

ATAACAGTTGGGAGAGATGGTTCATACATAGAT*CACGGTGAGAACGTTCTCTA*TTTTAAC

Fnr cre SinR

CATCTCAAATTATGGGCAAGAAAATAGAATAATAAAATGCGATAGTAATAGTTGCTAATA

CATAGGAGGAGTTAAAGTG *nheA*

>*hbl* 5'IGR *B. cereus* INRA C3

ATAATGATATTAGGATGTTTTGTGAAAAAATCAACAATATAACATATATTACTAAATATA

ResD

TCTACATTTTATGCAATTATACATAACTAAATAAAGGTAAAAAAGTATAAAAAGACCTAT

PlcR

TATATTATTCTATAAGTATTTTTTCTAAAATAAAATTTCTCGGTTGAGCTAAAATAGTTA

TTTTTAACCGTATACACATTAATTTGTAATCATTACAATTACAGAAATGAAATTTACGGA

TAACTATATTTGTATAATTTTTTTCGGTTCATATTTTTATTTAACAGTAAATTATCGATC

GTAATCGACAAAATTTTTCTATTTACGCATTAAAAATTTAATGTTTTAATGAACAACATA

ACTGGTATGACCAGACAGAAAGGA**TA**AGGTTACGCTAATAGGAATTATAGTGAAGTTGTA

Stab-SD

AGTAACATTATG*TTGAAAATATTTTT***C***GAA*TAGTCTATTTATTTACAAGAGGTCAAGAAT

ResD CodY

CAAGTTTGTAATAAACGTGTTCT**AA**GTTTCTGCATAAC*AAAAGTGAAGTTATTCCGCAAT*

SinR Fnr

AAAAGTATAAGCGATGTACAGTATAATTTTACCTTTTTTAGTCTAGTAAGGAGTTTGCTG

ATAAAACTAAGAGTAATATTATTAAATTTAAATTAAACAATGTTATGGCAAGATTAACTG

TAAGCATTGGTTTATTGATTCTCGCGGTTCTGTAAGTTAAACCGCAAT*TCTAGGGAAGAA*

SinR Fnr

*TTACACATTT*ACTATTCATAGGGTGCTTGATTCAAATATAGTTAATAAATTTTTGTTATT

TATATTTGAAAAAATAGAGTATCGAAGTGAGAGGTAAGTAAAATCCTTACATTCTATTAG

AAGTAAAAAATAACGATATTATCCTATCTGAAAGATTTCTCCTTCTTAATCAGTTTACAA

ResD

AGAGAGAGGTCATACAAGTTATATGAATTAAGTTATAAAAACAATGGTAAAGGAGTGTAC

GGAATG *hblC*

- PlcR consensus sequence: TATGNAN$_4$TNCATA (Gohar et al., 2008)
- ResD consensus sequences: TTGTN$_6$TTTNTN$_2$A or TTGTGAAN$_3$TTTN$_4$A or TTTGTGAAT (Geng et al., 2007)
- Stab-SD of *B. subtilis*: GAAAGGAGG (Agaisse and Lereclus, 1996)
- Fnr consensus sequence: TGTGAN$_6$TCACA (Esbelin et al., 2008)
- cre consensus sequence: Fig. 3 logo of the *B. cereus* cre site (van der Voort et al., 2008)

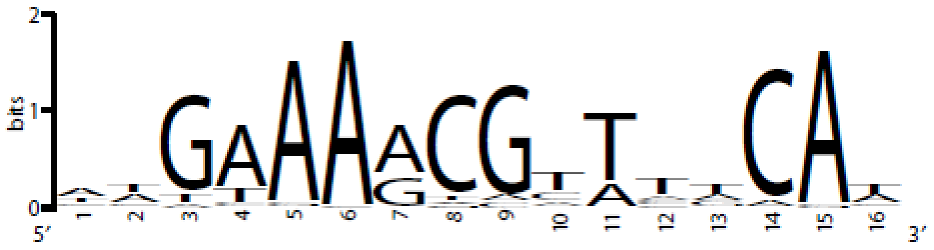

- SinR consensus sequence: GTTCTYT (Chu et al., 2006)
- CodY consensus sequence: Fig. 4 logo of the *B. subtilis* CodY binding sites (Belitsky and Sonenshein, 2013)

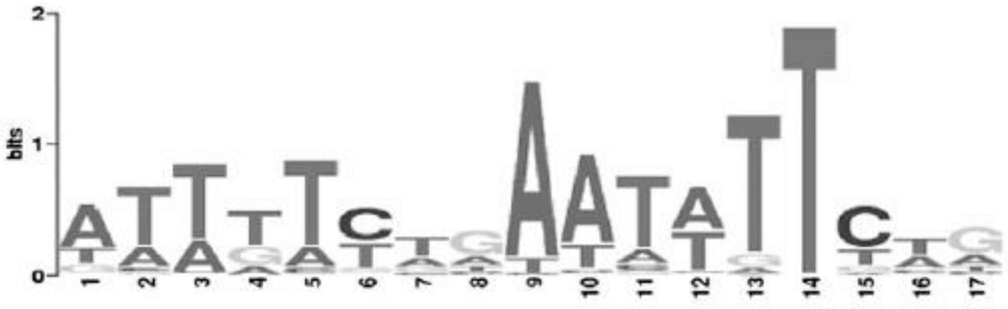

Fig. S2: Activity of the promoter regions in *B. cereus* INRA C3 in MOD minimal medium.

Growth and promoter activity kinetics of *B. cereus* INRA C3 pXen1 [P*nhe/lux*] and derivatives (**A**) and *B. cereus* INRA C3 pXen1 [P*hbl/lux*] and derivatives (**B**). Cell density was measured at an OD of 600 nm and bioluminescence intensity was recorded for 0.1 s at 490 nm with a luminescence microplate reader.

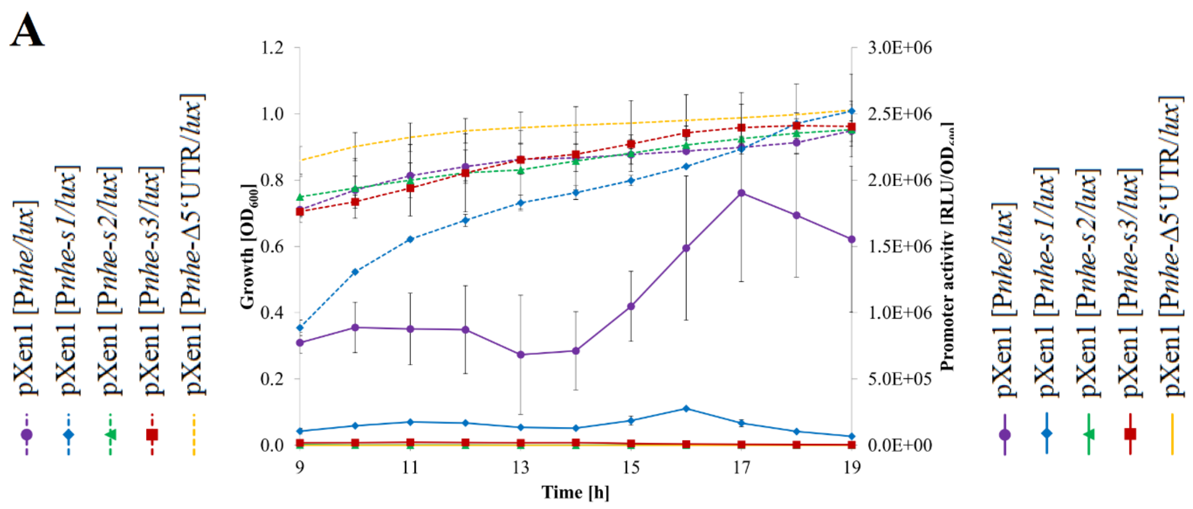

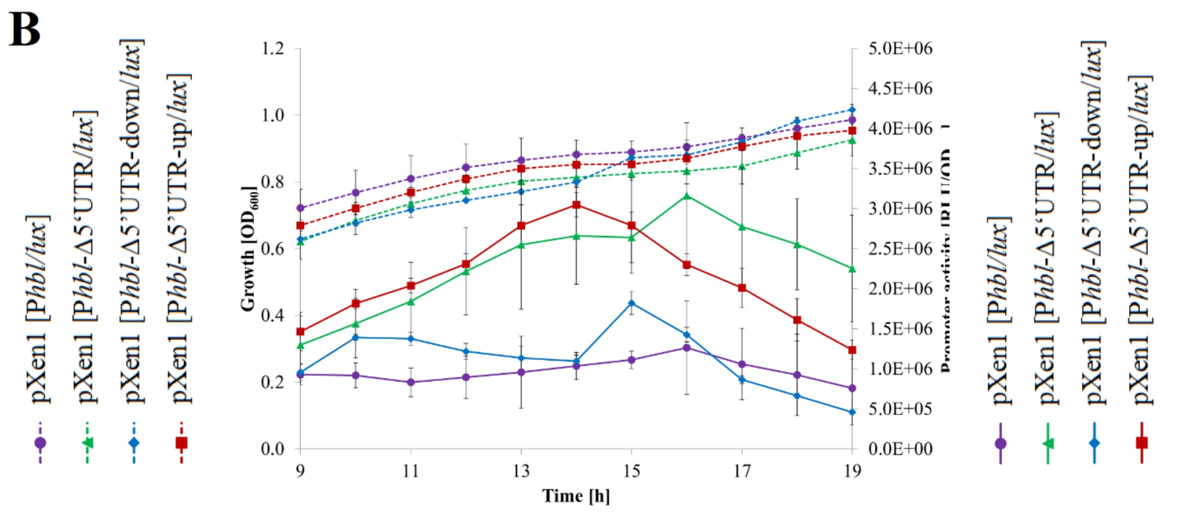

Fig. S3: Activity of the full-length enterotoxin promoter regions in *B. cereus* INRA C3 in media with differing amino acid availability.

Growth and promoter activity kinetics of *B. cereus* INRA C3 pXen1 [P*nhe/lux*] (**A**) and *B. cereus* INRA C3 pXen1 [P*hbl/lux*] (**B**) in defined MOD medium and MOD medium supplemented with either casamino acids (CAA) or tryptone. Cell density was measured at an OD of 600 nm and bioluminescence intensity was recorded for 0.1 s at 490 nm with a luminescence microplate reader. Growth and promoter activity kinetics in CGY medium are presented in Fig. 4A and B.

A

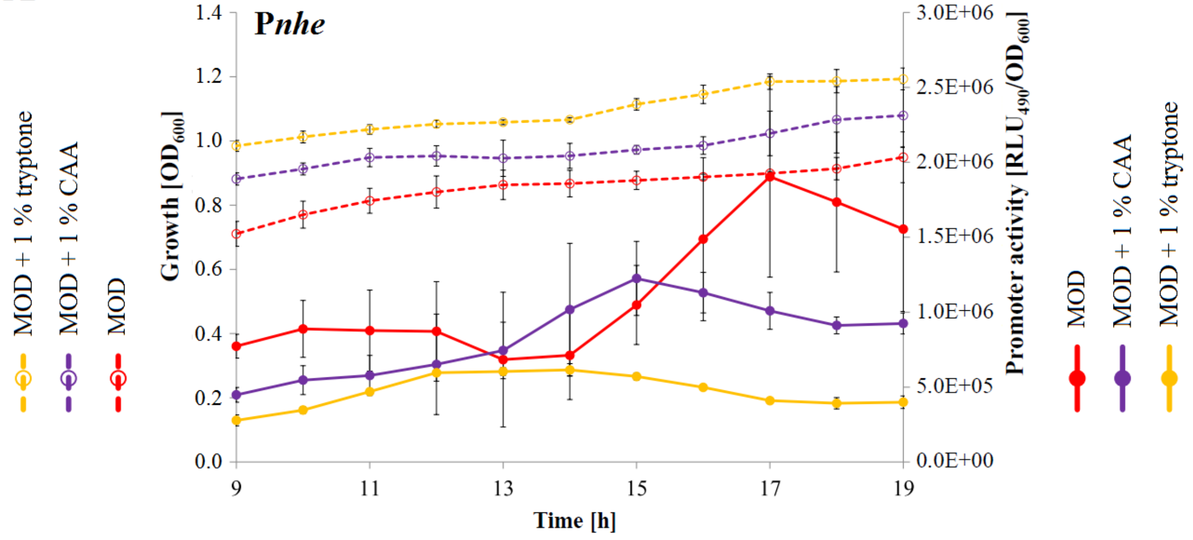

B

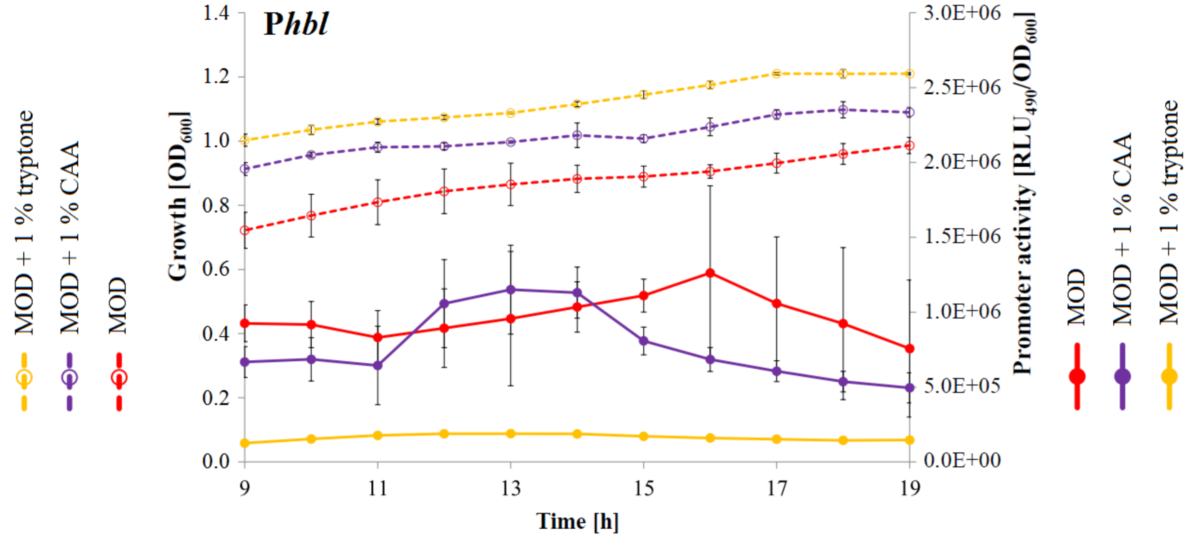

Fig. S4: Alignment of 5'IGR sequences of *nhe* of *B. cereus* ATCC 14579, *B. cereus* INRA C3, *B. cereus* F4810/72, *B. thuringiensis* Bt407 and *B. cytotoxicus* CVUAS 2833.

Potential CodY binding sites and translational starts are indicated in yellow. The first and second CodY binding sites are almost identical. However, the second binding site in *B. cytotoxicus* CVUAS 2833 is different, which might explain the varying binding affinity. A potential CodY binding site was located in P*nhe* of *B. cereus* F4810/72 (grey underlined), but no binding of CodY to P*nhe* F4810/72 could be detected (Frenzel et al., 2012). This site is not present in P*nhe* CVUAS 2833. P*nhe* INRA C3 and P*nhe* CVUAS 2833 were positive in electrophoretic mobility shift experiments.

The *nhe* 5'IGR of *B. cereus* INRA C3 (directly controlled by CodY) and F4810/72 (not directly controlled by CodY) share 89 % nucleotide sequence identity.

B. cereus INRA C3, *B. cereus* F4810/72 and *B. cytotoxicus* CVUAS 2833 were tested in gel mobility shift assays in this work and a previous study (Frenzel et al., 2012). The influence of CodY on virulence factor production via PlcR was analyzed in *B. cereus* ATCC 14579 and *B. thuringiensis* Bt407 in two previous studies (Lindbäck et al., 2012;Slamti et al., 2015).

```
CVUAS_2833      --TCTTTTTTAGTAGCTGCCTATAAGTCGGTGAAACCGACTTATAGGCAGCCTTTTTTAA
F4810/72        GAAGGTTTTTTAAGGATAGCTTTACGAA-----------AGTGAAGCTATCCT-------
INRA_C3         GAAGATTTTTTAAGGATAGTTTCATGAA-----------AATGAAGCTATCCT-------
ATCC_14579      GAAGATTTTTGAAGGATAGCTTCGTGAA-----------AATGAAGCTATCCT-------
Bt407           GAAGATTTTTGAAGGATAGCTTCGTGAA-----------AATGAAGCTATCCT-------
                     *****    * *   *    *               *  **  * ***


CVUAS_2833      TTTTATAAACAGAAGAGACTATGCACAATTACATATTCGCGAAATAATTATTTCAATATA
F4810/72        -----TTTTGTGTATACGCTATGCATAATTGCATACGAGTCTGAAAGATATATTGATATA
INRA_C3         -----TTTTGTGTATATGCTATGCATAATTGCATACGGGGTTAAAAATTATATCAATATA
ATCC_14579      -----TTTTGTGTATATGCTATGCATAATTGCATATGAGTTTAAAAATTATATCGATATA
Bt407           -----TTTTGTGTATATGCTATGCATAATTGCATATGGGTTTAAAAATTATATCGATATA
                     *     * * *  ******* **** ****   *    * *  *** *  *****


CVUAS_2833      ACACTATCTCTTGTGGAGAAACAAGCTTTTTTACTATTATGCATAGATATTTTAAATTGA
F4810/72        ATTTTTGTGTCTGTGAGTAAGTAGAGGTTTTTGTTGTGATATATGGATATTTCAATTGAA
INRA_C3         ATTTTACCGGTTGTTTGCAAGTAGTGTTTTCTGTTGTGATATGTAGATATTTCAATCGAT
ATCC_14579      ATTTTATCGGTTATTTGTAAGTAGTGTTTTCTGTTGTGATATGTAGATATTTCAATCAAT
Bt407           ATTTTATCTGTTATTTGCAAGTAGTGTTTTCTGTTGTGATATGTAGATATTTCAATCAAT
                *   *      * *    **  *    *** *  * * **   * ******* **


CVUAS_2833      AAAATCTTGATTACTTTATTTTAAAATAAGGAGATAAATCATTGGAGATAAAGGGAAATA
F4810/72        AAAGTAAAGTGGATTATATTTTAAAATAAAGACATGAAAGCTTGAAAATAAAGGGAATGA
INRA_C3         AAAGTGAATCGGATTATATTTTGAAATAAGGACATGAAAGCTTGAAAATAAAGGGAATGA
ATCC_14579      AAAGTGAATCGGATTATATTTTGAAATAAGGACATGAAAGCTTGAAAATAAAGGGAATGA
Bt407           AAAGTGAATCGGATTATATTTTGAAATAAGGACATGAAAGCTTGAAAATAAAGGGAATGA
                *** *       * * ****** ****** ** ** **   *** * **********  *
```

```
CVUAS_2833      AAATAGTTCTTTACGTTAA-TAAGGCGCTGTGTTTAAATAATTTTACAGTACAGAACTTG
F4810/72        AAATACTTCTTTACGTTATATAAATAGCTGTGTTTAAATGACCTTACAGTACAAAACTTA
INRA_C3         AAATACTTCTTTACGTTATATAAACAGCTGTGTTTAAATGACCTTACAGTACAAAACTTA
ATCC_14579      AAATACTTCTTTACGTTATATAAACAGCTGTGTTTAAATGACCTTACAGTACAAAACATA
Bt407           AAATACTTCTTTACGTTATATAAACAGCTGTGTTTAAATGACCTTACAGTACAAAACATA
                ***** ************  ***   ************* *  ********** *** *

CVUAS_2833      AATGAAAAACAAAAGATTGTT---------TTCTACGATTTTGCTAAAAATGAATAATAT
F4810/72        TATGAAAACAAAACGGAATTTCATATTTATGTATGTGAATTAGTTTAAAATGAATAATAT
INRA_C3         TATGAAAACAAAACGAAATTTCATATTTACGTATGTTGTTTGGTTTAAAATGAATAATAT
ATCC_14579      TATGAAAACAAAACGAAATTTCATATTTACGCATGTTGTTTAGTTTGAAATGAATAATAT
Bt407           TATGAAAACAAAACGAAATTTCATATTTACGCATGTTGTTTAGTTAAAAATGAATAATAT
                 *******  *** *    **            *     ** * *  *************

CVUAS_2833      TTTCATGTAATTTATGCAATGCGTAAATGGGCCCATTTTTCTATGCTTTTTAGTAAATTC
F4810/72        TTTTATGTAATATATTCACTGTATCAATGTGGGAAATAAGATGAAATAGAAAATTTATGC
INRA_C3         TTTTATGTAATATATTCACTGTGTGAATGTGGGAATTTCGATGAAATAGAAAATTTATAC
ATCC_14579      TTTTACGTAATATATTCACTATGTGAATGTGGGAATTTCTATAAAATAGAAAATTTATAC
Bt407           TTTTACGTAATATATTCACTGTGTGAATGTGGGAATTTCGATGAAATAGAAAATTTATAC
                *** * ***** *** ** *   * **** *   * *    *    *    * *  ** *

CVUAS_2833      AAGTTTGTGTTGTATTTTCAATTTCGTAAATAGGAAGAGAGTGCAA--GGGCTGAATGTT
F4810/72        AATGTTATTCATACTAACCACATATGAAAAGTTTTATAACGATAAGTAAGT-----ACTG
INRA_C3         AATATTATTCATACTAAACACATATGAAAAGTTTTATAACAATAAGTAAGT-----ACTG
ATCC_14579      AATATTATTCATACTAAACACATATGAAAAATAATAAAATGTTTTGTGACGATAAGTAAG
Bt407           AATATTATTCATACTAAACACATATGAAAAATAATAAAATGTTTTGTGACGATAAGTAAG
                **  ** *      *   **  *  * ***     * *

CVUAS_2833      AATTGTCCCTATTTAT---------------------GAAGGGTAACAACTGAATCTGTA
F4810/72        TAGCAATAAAATTTGGGAGAGATGGTTCGTGTGTGAATCGCGGTGAGAACGTTCTCTATT
INRA_C3         TAGCAATAACAGTTGGGAGAGATGGTTCATACATAGATCACGGTGAGAACGTTCTCTATT
ATCC_14579      TACTGTAGCAATACGAGAGAGGTGGTTCATACATGGATCACGGTGAGAACGTTCTCTATT
Bt407           TACTGTAGCAATACGAGAGAGGTGGTTCATACATGGATCACGGTGAGAACGTTCTCTATT
                 *        *                              *** * ***    *** *

CVUAS_2833      TAAAAAATAAATTGTT-----------------------AATT-----------------
F4810/72        TTAACCATCTCAAACTATGTGCAAGAAAATAGAATAATAAAATGCGATAGTAATAGTTAC
INRA_C3         TTAACCATCTCAAATTATGGGCAAGAAAATAGAATAATAAAATGCGATAGTAATAGTTGC
ATCC_14579      TTAATCATCTCAAATTATGGGCAAGAAAATAGAATAATAAAATGCGATAGTAATAGTTGC
Bt407           TTAATCATCTCAAATTATGGGCAAGAAAATAGAATAATAAAATGCGATAGTAATAGTTGC
                * **  **       *                       ** *

CVUAS_2833      TATACATTAGGAGGAATTAAAGTG             nheA
F4810/72        TAATACATAGGAGGAGTTAAAGTG             nheA
INRA_C3         TAATACATAGGAGGAGTTAAAGTG             nheA
ATCC_14579      TAATACATAGGAGGAGTTAAAGTG             nheA
Bt407           TAATACATAGGAGGAGTTAAAGTG             nheA
                **     ******** ********
```

Fig. S5: Alignment of 5'IGRs of the *hblCDAB* operon in *B. cereus* INRA C3, *B. cereus* ATCC 14579 and *B. thuringiensis* Bt407.

Translation start sites and potential CodY binding sites are highlighted. Yellow: Sites with ≥2 mismatches, red: sites with 1 mismatch in comparison to the consensus sequence (Belitsky and Sonenshein, 2013). The binding site to which CodY showed the strongest affinity in *in vitro* binding assays in *B. cereus* INRA C3 is underlined.

B. cereus INRA C3 was tested in gel mobility shift assays in this work. The influence of CodY on virulence factor production via PlcR was analyzed in *B. cereus* ATCC 14579 and *B. thuringiensis* Bt407 in two previous studies (Lindbäck et al., 2012;Slamti et al., 2015). *B. cereus* F4810/72 and *B. cytotoxicus* CVUAS 2833 do not contain *hbl*.

```
INRA_C3         ATAATGATATTAGGATGTTTTGTGAAAAAATCAACAATATAACATATATTACTAAATATA
ATCC_14579      ATAATGATATTAGGATGTTTTGTGAAAAAATCAACAATATAACATATATTACTAAATATA
Bt407           ATAATGATATTAGGATGTTTTGTGAAAAAATCAACAATATAACATATATTACTAAATATA
                ************************************************************

INRA_C3         TCTACATTTTATGCAATTATACATAACTAAATAAAGGTAAAAAAGTATAAAAAGACCTAT
ATCC_14579      TCTACATTTTATGCAATTATACATAACTAAATAAAGGTAAAAAAGTATAAAAAGACCTAT
Bt407           TCTACATTTTATGCAATTATACATAACTAAATAAAGGTAAAAAAGTATAAAAAGACCTAT
                ************************************************************

INRA_C3         TATATTATTCTATAAGTATTTTTTCTAAAATAAAATTTCTCGGTTGAGCTAAAATAGTTA
ATCC_14579      TATATTATTCTATAAGTATTTTTTCTAAAATAAAATTTCTCGGTTGAGCTAAAATAGTTA
Bt407           TATATTATTCTATAAGTATTTTTTCTAAAATAAAATTTCTTGGTTGAGCTAAAATAGTTA
                **************************************** *******************

INRA_C3         TTTTTAACCGTATACACATTAATTTGTAATCATTACAATTACAGAAATGAAATTTACGGA
ATCC_14579      TTTTTAACCGTATACACATTAATTTGTAATCATTACAATTACAGAAATGAAATTTACGGA
Bt407           TTTTTAACCGTATACACATTAATTTGTAATCATTACAATTACAGAAATGAAATTTACGGA
                ************************************************************

INRA_C3         TAACTATATTTGTATAATTTTTTTCGGTTCATATTTTTATTTAACAGTAAATTATCGATC
ATCC_14579      TAACTATATTTGTATAATTTTTTTCGGTTCATATTTTTATTTAACAGTAAATTATCGATC
Bt407           TAACTATATTTATATAATTT-TTTCGGTTCATATTTTTATTTAACAGTAAATTATCGATC
                *********** ******** ***************************************

INRA_C3         GTAATCGACAAAATTTTTCTATTTACGCATTAAAAATTTAATGTTTTAATGAACAACATA
ATCC_14579      GTAATCGACAAAATTTTTCTATTTACGCATTAAAAATTTAATGTTTTAATGAACAACATA
Bt407           GTAATCGACAAAATTTTTCTATTTACGCATTAAAAATTTAATGTTTTAATGAACAACATA
                ************************************************************

INRA_C3         ACTGGTATGACCAGACAGAAAGGATAAGGTTACGCTAATAGGAATTATAGTGAAGTTGTA
ATCC_14579      ACTGGTATGACCAGACAGAAAGGATAAGGTTACGCTAATAGGAATTATAGTGAAGTTGTA
Bt407           ACTGGTATGACCAGACAGAAAGGATAAGGTTACGCTAATAGGAATTATAGTGAAGTTGTA
                ************************************************************
```

```
INRA_C3         AGTAACATTATGTTGAAAATAT-TTTTCGAATAGTCTATTTATTTACAAGAGGTCAAGAA
ATCC_14579      AGTAACATTATGTTGAAAATAT-TTTTCGAATAGTCTATTTATTTACAAGAGGTCAAGAA
Bt407           AGTAACATTATGTCGTTAATAATTTTATGAATAGTCAATTTATTTACAAGAGGTCAAGAA
                ************* *  ****  ***  ******** ***********************

INRA_C3         TCAAGTTTGTAATAAACGTGTTCTAAGTTTCTGCATAACAAAAGTGAAGTTATTCCGCAA
ATCC_14579      TCAAGTTTGTAATAAACGTGTTCTAAGTTTCTGCATAACAAAAGTGAAGTTATTCCGCAA
Bt407           TCAAATTTGTAATAAACCTGTTCTAAGTTTCTGCATAACAAAAGTGAAGTTATTCCGCAA
                **** ************ ******************************************

INRA_C3         TAAAAGTATAAGCGATGTACAGTATAATTTTACCTTTTTTAGTCTAGTAAGGAGTTTGCT
ATCC_14579      TAAAAGTATAAGCGATGTACAGTATAATTTTACCTTTTTTAGTCTAGTAAGGAGTTTGCT
Bt407           TAAAAGTATAAGCGATGTACAGTATAATTTCACCTTTTGTAGTCTAGTAAGGAGCTTGCT
                ****************************** ******* *************** *****

INRA_C3         GATAAAACTAAGAGTAATATTATTAAATTTAAATTAAACAATGTTATGGCAAGATTAACT
ATCC_14579      GATAAAACTAAGAGTAATATTATTAAATTTAAATTAAACAATGTTATGGCAAGATTAACT
Bt407           GATAAAACTAAGAGTAATATTATTAAATTTAAATTAAACAATGTTATGACAAGATTAACT
                ************************************************ ***********

INRA_C3         GTAAGCATTGGTTTATTGATTCTCGCGGTTCTGTAAGTTAAACCGCAATTCTAGGGAAGA
ATCC_14579      GTAAGCATTGGTTTATTGATTCTCGCGGTTCTGTAAGTTAAACCGCAATTCTAGGGAAGA
Bt407           GTAAGCATTGGTTTATTGATTCTCGCAGTTCTGTAAGTTAAACCGCAATTCTAGGGAAGA
                ************************** *********************************

INRA_C3         ATTACACATTTACTATTCATAGGGTGCTTGATTCAAATATAGTTAATAAATTTTTGTTAT
ATCC_14579      ATTACACATTTACTATTCATAGGGTGCTTGATTCAAATATAGTTAATAAATTTTTGTTAT
Bt407           ATTACTCATTTACTATTCATAGGGTGCTTGATTCAAATATAGTTAATAAATTTTTGTTAT
                ***** ******************************************************

INRA_C3         TTATATTTGAAAAAATAGAGTATCGAAGTGAGAGGTAAGTAAAATCCTTACATTCTATTA
ATCC_14579      TTATATTTGAAAAAATAGAGTATCGAAGTGAGAGGTAAGTAAAATCCTTACATTCTATTA
Bt407           TTATATTTGAAAAAATAAAGTATCGAAGTGAGAAGTAAGTAAAATCCTTACATCCTATTA
                ***************** *************** ******************* ******

INRA_C3         GAAGTAAAAAATAACGATATTATCCTATCTGAAAGATTTCTCCTTCTTAATCAGTTTACA
ATCC_14579      GAAGTAAAAAATAACGATATTATCCTATCTGAAAGATTTCTCCTTCTTAATCAGTTTACA
Bt407           GAAGTAAAAAATAACGATATTATCCTATCTGAAAGATTTCTCCTTCTTAATCAGTTTACA
                ************************************************************

INRA_C3         AAGAGAGAGGTCATACAAGTTATATGAATTAAGTTATAAAAACAATGGTAAAGGAGTGTA
ATCC_14579      AAGAGAGAGGTCATACAAGTTATATGAATTAAGTTATAAAAACAATGGTAAAGGAGTGTA
Bt407           AAGAGAGAGGTCGTACAAGTTATATGAATTAAGTTATAAAAACAATGGTAAAGGAGTGTA
                ************ ***********************************************

INRA_C3         CGGAATG         hblC
ATCC_14579      CGGAATG         hblC
Bt407           CGGAATG         hblC
                *******
```

P*hbl* parts of *B. cereus* INRA C3 tested in gel mobility shift experiments:

>1

AATAATGATATTAGGATGTTTTGTGAAAAAATCAACAATATAACATATATTACTAAATAT
ATCTACATTTTATGCAATTATACATAACTAAATAAAGGTAAAAAAGTATAAAAAGACCTA
TTATATTATTCTATAAGTATTTTTTCTAAAATAAAATTTCTCGGTTGAGCTAAAATAGTT
ATTTTTAACCGTATACACATTAATTTGTAATCATTACAATTACAGAAATGAAATTTACGG
ATAACTATATTTGTATAATTTTTTTCGGTTCATATTTTTATTTAACAGTAAATTATCGAT
CGTAATC

>2

AAATTATCGATCGTAATCGACAAAATTTTTCTATTTACGCATTAAAAATTTAATGTTTTA
ATGAACAACATAACTGGTATGACCAGACAGAAAGGATAAGGTTACGCTAATAGGAATTAT
AGTGAAGTTGTAAGTAACATTATGTTGAAAATATTTTTCGAATAGTCTATTTATTTACAA
GAGGTCAAGAATCAAGTTTGTAATAAACGTGTTCTAAGTTTCTGCATAACAAAAGTGAAG
TTATTCCGCAATAAAAGTATAAGCGATGTACAGTATAATTTTACCTTTTTTAGTCTAGTA
AGGAGTTTGCTGA

>3

TCTAGTAAGGAGTTTGCTGATAAAACTAAGAGTAATATTATTAAATTTAAATTAAACAAT
GTTATGGCAAGATTAACTGTAAGCATTGGTTTATTGATTCTCGCGGTTCTGTAAGTTAAA
CCGCAATTCTAGGGAAGAATTACACATTTACTATTCATAGGGTGCTTGATTCAAATATAG
TTAATAAATTTTTGTTATTTATATTTGAAAAAATAGAGTATCGAAGTGAGAGGTAAGTAA
AATCCTTACATTCTATTAGAAGTAAAAAATAACGATATTATCCTATCTGAAAGATTTCTC
CTTCTTAATCAGTTTACAAAGAGAGAGGTCATACAAGTTATATGAATTAAGTTATAAAAA
CAATGGTAAAGGAGTGTACGGAAT

>4

CAAGTTTGTAATAAACGTGTTCTAAGTTTCTGCATAACAAAAGTGAAGTTATTCCGCAAT
AAAAGTATAAGCGATGTACAGTATAATTTTACCTTTTTTAGTCTAGTAAGGAGTTTGCTG
ATAAAACTAAGAGTAATATTATTAAATTTAAATTAAACAATGTTATGGCAAGATTAACTG
TAAGCATTGGTTTATTGATTCTCGCGGTTCTGTAAGTTAAACCGCAATTCTAGGGAAGAA
TTACACATTTACTATTCATAGGGTGCTTGATTCAAATATAGTTAATAAATTTTTGTTATT
TATATTTGAAAAAATAGAGTATCGAAGTGAGAG

>5

GTATACACATTAATTTGTAATCATTACAATTACAGAAATGAAATTTACGGATAACTATAT
TTGTATAATTTTTTTCGGTTCATATTTTTATTTAACAGTAAATTATCGATCGTAATCGAC
AAAATTTTTCTATTTACGCATTAAAAATTTAATGTTTTAATGAACAACATAACTGGTATG
ACCAGACAGAAAGGATAAGGTTACGCTAATAGGAATTATAGTGAAGTTGTAAGTAACATT
ATGTTGAAAATATTTTTCGAATAGTCTATTTATTTACAAGAGGTCAAGAATCAAGTTTGT
AATAAACGTGTTC

Fig. S6: Alignment of CodY amino acid sequences.

Amino acid sequences from *B. subtilis* subsp. subtilis 6051-HGW, *B. cytotoxicus* CVUAS 2833, *B. cereus* INRA C3 and *B. cereus* F4810/72 were aligned and functional domains (Levdikov et al., 2006;Stenz et al., 2011) are marked. **Δ:** important for isoleucine ligand binding, **°:** important for dimer formation, **•**: important for DNA-binding.

B. subtilis CodY shares an overall amino acid sequence identity of ~80 % with *B. cereus* CodY: 81 % F4810/72, 82 % INRA C3, 83 % CVUAS 2833. Segments β2 to β3 and β3 to β4 are responsible for BCAA ligand binding. Segments β8 to α9 (aa 203 – 226) are the HTH-domain. N-terminal cofactor binding domain (variable) and C-terminal DNA-binding domain (highly conserved) are present within a single polypeptide chain. N-terminal GAF (cGMP-stimulated phosphodiesterases, adenylate cyclases and a bacterial transcription regulator FhlA, α1 – α5) domain is responsible for BCAA-binding and could also provide GTP-binding. Differences between strains are highlighted in blue. All differences except 251 are located in the N-terminal region. Exchanges at positions 94, 133, 147 and 251 lead to a change in amino acid polarity and charge.

```
                          N-terminus
                         1                  20                  40                  60
                                 α1                 α2         β1        β2
                           _______________      ____________  ______    _____       _
B. subtilis 6051         MALLQKTRIINSMLQAAAGKPVNFKEMAETLRDVIDSNIFVVSRRGKLLGYSINQQIEND
Bcyt CVUAS 2833          MELLAKTRKLNALLQSAAGKPVNFREMSDTMCEVIEANVFVVSRRGKLLGYAIHQQIENE
Bc INRA C3               MELLAKTRKLNALLQSAAGKPVNFREMSDTMCEVIEANVFVVSRRGKLLGYAIHQQIENE
Bc F4810/72              MELLAKTRKLNALLQSAAGKPVNFREMSDTMCEVIEANVFVVSRRGKLLGYAIHQQIENE
                         * ** ***  *  ** ******** **  *   **  * ************ * *****
                           ∘∘  ∘∘  ∘  ∘∘

                         61                 80                  100                 120
                           α3          α4         β3            η1          β4
                         ________    _______     _____          ___      _________
B. subtilis 6051         RMKKMLEDRQFPEEYTKNLFNVPETSSNLDINSEYTAFPVENRDLFQAGLTTIVPIIGGG
Bcyt CVUAS 2833          RMKQMLAERQFPEEYTQNLFNVTETSSNLDVNSEYTAFPVENKDLFGQGLTTIVPIVGGG
Bc INRA C3               RMKQMLAERQFPEEYTQSLFNITETSSNLDVNSAYTAFPVENRELFGQGLTTIVPIVGGG
Bc F4810/72              RMKQMLAERQFPEEYTQSLFNITETSSNLDVNSAYTAFPVENKELFGQGLTTIVPIVGGG
                         *** **  ********  ***  ******* ** ********  **  ******** ***
                         ΔΔ  Δ     ΔΔ  Δ                      ΔΔ

                         121                140                 160                 180
                              β5                 α5                          α6
                          __________     __________________              __________
B. subtilis 6051         ERLGTLILSRLQDQFNDDDLILAEYGATVVGMEILREKAEEIEEEARSKAVVQMAISSLS
Bcyt CVUAS 2833          ERLGTLVLARLGREFLDDDLILAEYSATVVGMEILREKAEEIEEEARSKAVVQMAISSLS
Bc INRA C3               ERLGTLVLARLGQEFLDDDLILAEYSSTVVGMEILREKAEEIEEEARSKAVVQMAISSLS
Bc F4810/72              ERLGTLVLARLGQEFLDDDLILAEYSSTVVGMEILREKAEEIEEEARSKAVVQMAISSLS
                         ****** * **   * *********  *********************************
                                         ∘  ∘∘  ∘∘  ∘∘  ∘∘ ∘

                         181                200                 220                 240
                            α7             β6    α8             α9
                         __________       ____ _______      ___________
B. subtilis 6051         YSELEAIEHIFEELDGNEGLLVASKIADRVGITRSVIVNALRKLESAGVIESRSLGMKGT
Bcyt CVUAS 2833          YSELEAIEHIFEELNGTEGLLVASKIADRVGITRSVIVNALRKLESAGVIESRSLGMKGT
Bc INRA C3               YSELEAIEHIFEELNGTEGLLVASKIADRVGITRSVIVNALRKLESAGVIESRSLGMKGT
Bc F4810/72              YSELEAIEHIFEELNGTEGLLVASKIADRVGITRSVIVNALRKLESAGVIESRSLGMKGT
                         ************** * *******************************************
                                                   ••  •  ••

                                              C-terminus
                         241
                                   α10
                                 _______
B. subtilis 6051         YIKVLNNKFLIELENLKSH
Bcyt CVUAS 2833          YIKVLNDKFLHELAKLKTN
Bc INRA C3               YIKVLNDKFLQELAKLKTN
Bc F4810/72              YIKVLNDKFLHELAKLKTN
                         ****** *** **  **
```

Fig. S7: Activity of the full-length P*hbl* and P*hbl*-Δ5'UTR in *B. cereus* INRA C3 in media with differing amino acid availability.

Cell density was measured at an OD of 600 nm and bioluminescence intensity was recorded for 0.1 s at 490 nm with a luminescence microplate reader. Promoter activities were determined in triplicates and peak activities were compared. Luminescence signals generated by an active transcription of the *lux* genes are proportional to the activity of the promoter region tested. Maximum promoter activity was detected as follows (hours after inoculation): MOD: 16 h, MOD + 1 % tryptone: 13 h, MOD + 1 % casamino acids (CAA): 13 h, CGY: 18 h.

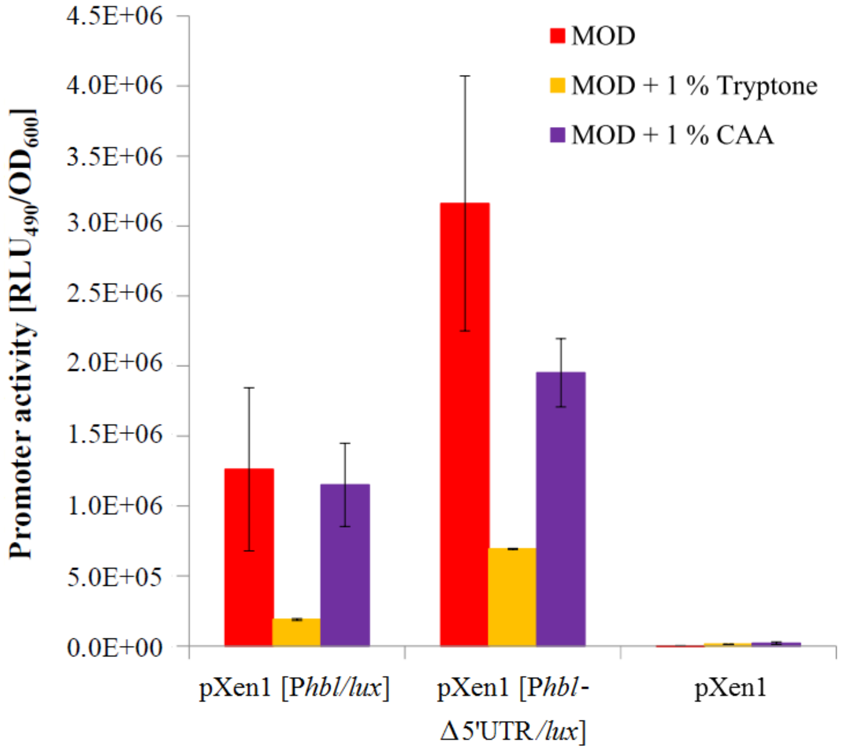

References

Agaisse, H., and Lereclus, D. (1996). STAB-SD: a Shine-Dalgarno sequence in the 5' untranslated region is a determinant of mRNA stability. *Mol Microbiol* 20**,** 633-643.

Belitsky, B.R., and Sonenshein, A.L. (2013). Genome-wide identification of *Bacillus subtilis* CodY-binding sites at single-nucleotide resolution. *Proc Natl Acad Sci U S A* 110**,** 7026-7031.

Chu, F., Kearns, D.B., Branda, S.S., Kolter, R., and Losick, R. (2006). Targets of the master regulator of biofilm formation in *Bacillus subtilis*. *Mol Microbiol* 59**,** 1216-1228.

Esbelin, J., Jouanneau, Y., Armengaud, J., and Duport, C. (2008). ApoFnr binds as a monomer to promoters regulating the expression of enterotoxin genes of *Bacillus cereus*. *J Bacteriol* 190**,** 4242-4251.

Frenzel, E., Doll, V., Pauthner, M., Lucking, G., Scherer, S., and Ehling-Schulz, M. (2012). CodY orchestrates the expression of virulence determinants in emetic *Bacillus cereus* by impacting key regulatory circuits. *Mol Microbiol* 85**,** 67-88.

Geng, H., Zhu, Y., Mullen, K., Zuber, C.S., and Nakano, M.M. (2007). Characterization of ResDE-dependent *fnr* transcription in *Bacillus subtilis*. *J Bacteriol* 189**,** 1745-1755.

Gohar, M., Faegri, K., Perchat, S., Ravnum, S., Okstad, O.A., Gominet, M., Kolsto, A.B., and Lereclus, D. (2008). The PlcR virulence regulon of *Bacillus cereus*. *PLoS One* 3**,** e2793.

Levdikov, V.M., Blagova, E., Joseph, P., Sonenshein, A.L., and Wilkinson, A.J. (2006). The structure of CodY, a GTP- and isoleucine-responsive regulator of stationary phase and virulence in gram-positive bacteria. *J Biol Chem* 281**,** 11366-11373.

Lindbäck, T., Mols, M., Basset, C., Granum, P.E., Kuipers, O.P., and Kovacs, A.T. (2012). CodY, a pleiotropic regulator, influences multicellular behaviour and efficient production of virulence factors in *Bacillus cereus*. *Environ Microbiol* 14**,** 2233-2246.

Martineau, F., Picard, F.J., Roy, P.H., Ouellette, M., and Bergeron, M.G. (1996). Species-specific and ubiquitous DNA-based assays for rapid identification of *Staphylococcus epidermidis*. *J Clin Microbiol* 34**,** 2888-2893.

Slamti, L., Lemy, C., Henry, C., Guillot, A., Huillet, E., and Lereclus, D. (2015). CodY Regulates the Activity of the Virulence Quorum Sensor PlcR by Controlling the Import of the Signaling Peptide PapR in *Bacillus thuringiensis*. *Front Microbiol* 6**,** 1501.

Stenz, L., Francois, P., Whiteson, K., Wolz, C., Linder, P., and Schrenzel, J. (2011). The CodY pleiotropic repressor controls virulence in gram-positive pathogens. *FEMS Immunol Med Microbiol* 62**,** 123-139.

van Der Voort, M., Kuipers, O.P., Buist, G., De Vos, W.M., and Abee, T. (2008). Assessment of CcpA-mediated catabolite control of gene expression in *Bacillus cereus* ATCC 14579. *BMC Microbiol* 8**,** 62.
